# Host–guest chemistry on living cells enabling recyclable photobiocatalytic cascade†

**DOI:** 10.1039/d4sc06508e

**Published:** 2025-02-06

**Authors:** Jiaheng Zhang, Vasco F. Batista, René Hübner, Henrik Karring, Changzhu Wu

**Affiliations:** a Department of Physics, Chemistry and Pharmacy, University of Southern Denmark Campusvej 55 5230 Odense Denmark wu@sdu.dk; b Danish Institute for Advanced Study (DIAS), University of Southern Denmark Campusvej 55 5230 Odense Denmark; c Helmholtz-Zentrum Dresden – Rossendorf (HZDR), Institute of Ion Beam Physics and Materials Research Bautzner Landstrasse 400 01328 Dresden Germany; d Department of Green Technology, University of Southern Denmark Campusvej 55 5230 Odense Denmark

## Abstract

Combining chemical and whole-cell catalysts enables sustainable chemoenzymatic cascade reactions. However, their traditional combination faces challenges in catalyst recycling and maintaining cell viability. Here, we introduce a supramolecular host–guest strategy that efficiently attaches photocatalysts to bacterial cells, facilitating recyclable photobiocatalysis. This method involves attaching a cationic polyethylenimine (PEI) polymer, functionalized with β-cyclodextrin (β-CD), to *E. coli* cells. The polymer attachment is biocompatible and protective, safeguarding the cells from harsh conditions such as UV radiation and organic solvents, without causing cell death. Additionally, the presence of β-CD imparts a plug-and-play capability to the cells, enabling the straightforward integration of guest photocatalysts – specifically anthraquinone – onto the cell surface through host–guest interactions. This effective combination of cellular and chemical catalysts promotes efficient photobiocatalytic cascades and supports the photocatalyst's recycling and reuse. This supramolecular system thus represents a promising platform for advancing photobiocatalysis in cascade synthesis.

## Abstract 2

Host–guest chemistry on the surface of bacterial cells containing a cyclodextrin-functionalized polymer enables the biocompatible combination of photo and biocatalysts as recyclable systems for cascade reactions.

## Graphical abstract

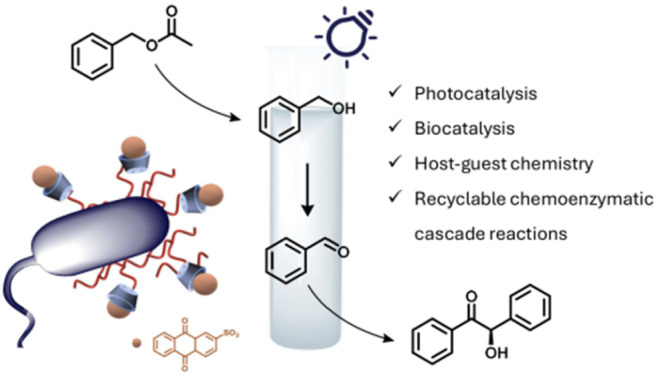

## Introduction

Biocatalysis, using enzymes and whole cells, presents a practical and sustainable alternative to traditional organo- and metal-catalysis methods.^1,2^ This approach has evolved into a widely adopted and potent strategy for chemical synthesis.^3–5^ Nevertheless, individual natural biocatalysts are inherently limited by their naturally available properties and single-step reactivity, rendering them challenging for multistep industrial synthesis.^6,7^

An elegant solution to this limitation involves combining biocatalysts with synthetic chemical catalysts to create chemoenzymatic reactions.^8,9^ This combination can simplify processes and separation, minimize side-product formation, and drive reaction equilibria towards more favorable products.^10–14^ Significant success has been achieved in this area, particularly in kinetic dynamic resolutions of racemic alcohols and amines, as well as in cofactor regeneration.^15–19^ Additionally, photocatalysts have been successfully integrated with enzymes for photo-enzymatic reactions, facilitating various value-added syntheses.^20–23^

Despite these advancements, the field of chemoenzymatic cascades faces substantial challenges. One of the primary hurdles is catalyst recycling.^24,25^ While dual-catalyst systems eliminate intermediate separations, they complicate the reuse of both catalysts within a single system, especially when homogeneous metal- and organo-catalysts are employed.^26^ Another significant challenge is the issue of compatibility, as chemical and biological catalysts typically operate in different media and at varying temperatures, potentially deactivating each other.^27,28^ These constraints have limited chemoenzymatic reactions to a narrow range of enzymes and chemical catalysts to date.

**Scheme 1:**
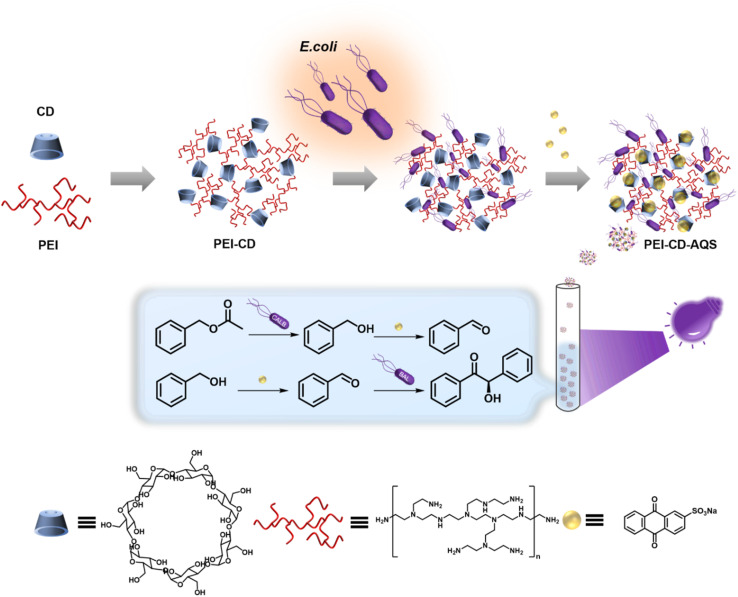
Host–guest chemistry on *E. coli* cells for cascade photobiocatalysis (CD: cyclodextrin; PEI: polyethylenimine; *E. coli*: *Escherichia coli*; AQS: anthraquinone-2-sulfonic acid sodium salt).

To address these challenges, in this study, we introduce a supramolecular coating approach to anchor chemical catalysts to whole-cell catalysts, resulting in facile, recyclable photobiocatalytic systems. This coating is achieved by attaching a supramolecular polymer, specifically β-cyclodextrin-containing polyethylenimine (PEI-CD), to *Escherichia coli* (*E. coli*) cells. The PEI-CD polymers offer a dual function: they act as a protective coat against harsh reaction conditions and provide a supramolecular framework that facilitates the easy bacterial recycling and the introduction of guest catalysts for cascade reactions. As a proof-of-concept, we employed the classic photocatalyst, anthraquinone-2-sulfonic acid sodium salt (AQS), to interact with *E. coli* cells, which overexpressed benzaldehyde lyase or *Candida Antarctica* lipase B (CalB), while coated with PEI-CD. Our results demonstrate that the supramolecular coating enables efficient host–guest chemistry between cells and AQS, creating a catalyst-cell complex. Remarkably, this combination enables two different cascade transformations that are not only highly efficient but also recyclable. Thus, our approach offers a conceptual platform for designing recyclable chemoenzymatic cascade reactions and is envisioned to be applicable in various green chemistry and advanced synthesis contexts (Scheme 1).

## Results and discussion

### Supramolecular coating on cells

The supramolecular coating was achieved through a two-step process. Initially, we synthesized PEI-CD *via* amide coupling. The successful synthesis of the desired product was confirmed by ^1^H NMR spectroscopy (Fig. S1†). The appearance of new signals corresponding to the methylene protons of PEI at *δ* 2.4–2.9 ppm confirmed the functionalization of PEI with β-CD. The average molar ratio of PEI to β-CD was calculated as approximately 6 : 1 by integrating the characteristic signals corresponding to the PEI methylene protons and anomeric protons of β-CD as follows: (area of PEI signal between 2.2–2.8/average number of methylene protons per PEI molecule)/(area of CD signal between 4.7–5.1/number of anomeric protons per CD molecule). In the second step, we coated *E. coli* cells by exploiting the electrostatic interaction between the negatively charged cells and the positively charged PEI-CD. After the coating, the cells were washed several times to remove the unbound polymers.

**Fig. 1:**
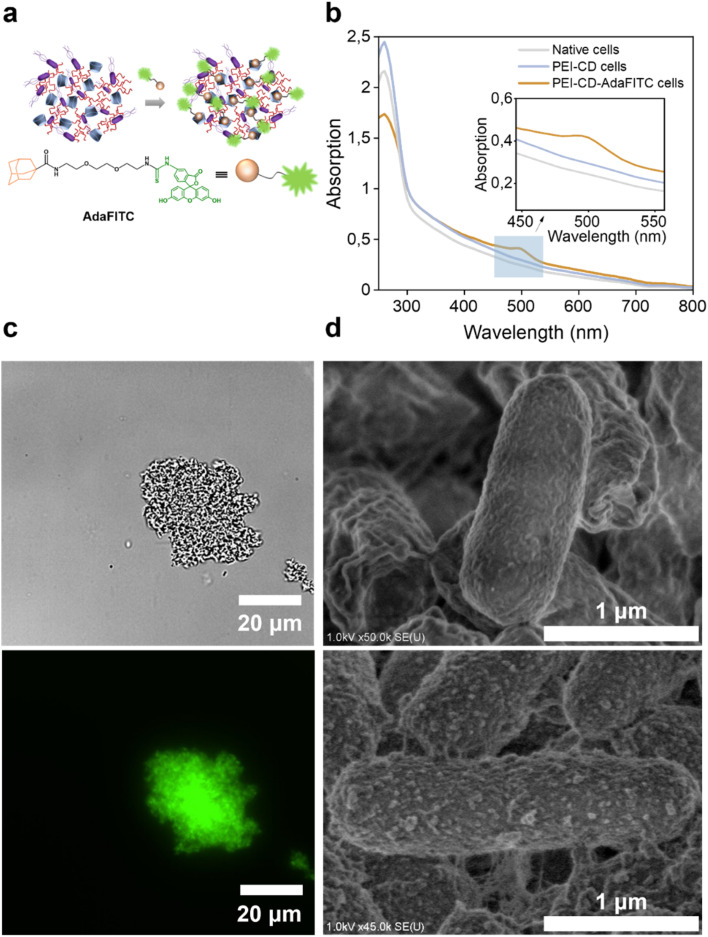
Characterization of PEI-CD and PEI-CD-AdaFITC cells. (a) Formation of PEI-CD-AdaFITC cells host–guest complexes. (b) UV-vis spectrum of native, PEI-CD, and PEI-CD-AdaFITC cells. (c) Bright field (top) and fluorescence microscopy (bottom) images of PEI-CD-AdaFITC cells. (d) SEM images of native (top) and PEI-CD (bottom) cells.

The success of the coating was confirmed by thermogravimetric analysis (TGA), which showed an increased mass loss for the coated cells compared to the native cells (Fig. S2 and S3†). Further confirmation was obtained by observing the interaction of these cells with a synthetic fluorescein isothiocyanate (FITC) dye containing an adamantane moiety (AdaFITC) (Fig. 1a). Adamantane is a well-known guest molecule that can be easily encapsulated into the hydrophobic cavity of CD, making it an effective marker for the presence of PEI-CD.^29–31^ The UV-vis spectrum of PEI-CD-AdaFITC-coated cells thoroughly washed with KPi buffer (Fig. 1b) exhibited a distinct peak at 488 nm, characteristic of FITC molecules. This peak was absent in the UV-vis spectra of native and PEI-CD cells, confirming the efficient encapsulation of AdaFITC within the CD cavity. Fluorescence microscopy (Fig. S6†) demonstrated the presence of well-dispersed uncoated cells in the bright field, with no fluorescence observed in the fluorescent channel. This finding is consistent with the results of cells treated with AdaFITC and washed, thus excluding the possibility of fluorescence from AdaFITC binding to cells. Interestingly, the PEI-CD-coated cells (Fig. 1c) formed clusters, likely due to random interactions between PEI-CD and the bacterial surface.^32–34^ These clusters exhibited strong green fluorescence even after several washing steps, further confirming the successful attachment of PEI-CD to the bacterial surface and the formation of host–guest complexes with AdaFITC moieties. Further testing revealed that this cluster formation depended on the concentration of PEI-CD, with significant aggregation already observed at a PEI-CD concentration of 1.6 mg mL^−1^ (Fig. S7†).

Next, the morphology of the cell surface was analyzed by scanning electron microscopy (SEM). As shown in Fig. 1d, native cells presented a relatively smooth and natural cellular surface. In contrast, PEI-CD-coated cells exhibited a rougher surface, suggesting the bacterial surface was adhered to and covered by the material. Furthermore, transmission electron microscopy (TEM) provided detailed insights into the morphological changes induced by the coating process. TEM images revealed clear differences between native and PEI-CD-modified cells (Fig. S8†). Native cells showed a well-defined cell membrane, while PEI-CD-modified cells exhibited significant structural changes. As a result, electron microscopy studies provided evidence of nanoscale modifications and interactions induced by the PEI-CD coating on the bacterial surface.

### Biocompatible coating

**Fig. 2:**
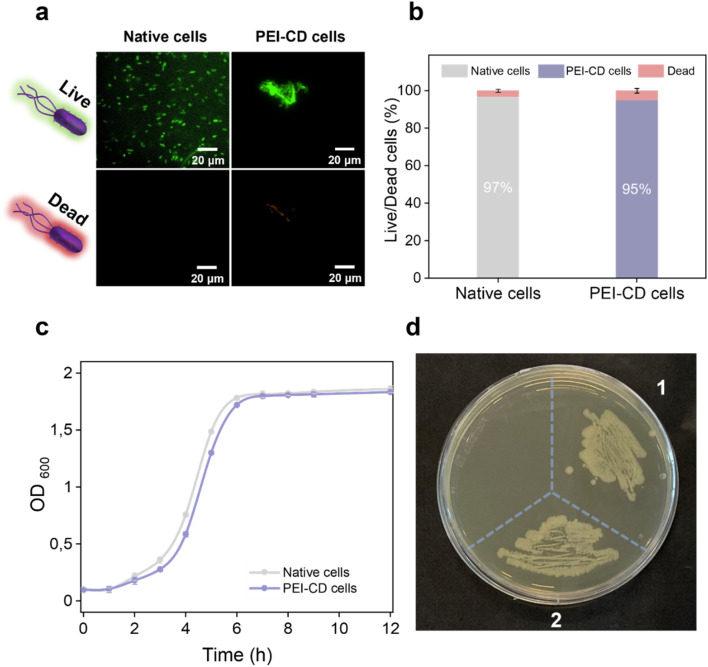
Biocompatibility of PEI-CD-coated bacterial cells. (a) Fluorescence microscopy images and (b) data analysis of the live/dead assay of bacterial cells. (c) Growth curve of native (grey curve) and PEI-CD-coated (blue curve) *E. coli* cells. (d) Plating assay of native (1) and PEI-CD-coated (2) cells.

Given the presence of positively charged polymers used for cell coating, we assessed their cytotoxicity using a live/dead assay. This assay employed two fluorescent dyes: SYTO™ 9, which stains live cells green, and propidium iodide, which stains dead cells red. Fresh, live cells collected at OD_600_ = 0.6 served as the control group. As shown in Fig. 2a, approximately 97% of these control cells exhibited green fluorescence. Notably, after PEI-CD attachment, approximately 95% of the cells retained green fluorescence, indicating that PEI-CD did not cause significant damage to the bacteria and exhibited excellent biocompatibility. Furthermore, re-culture studies revealed similar results (Fig. 2c), with the PEI-CD-coated cells displaying growth similar to the control group. Additionally, in a plating assay, PEI-CD-coated bacterial cells demonstrated excellent proliferative ability (Fig. 2d). Previous reports on covalent bacterial surface modification also suggest that the polymer may be distributed between the proliferating cells upon division.^35^ Despite the general cytotoxicity associated with positively charged polymers, our results confirmed the biocompatibility of PEI-CD. This biocompatibility might be attributed to the low concentration of PEI-CD used or the modification with β-CD, which may shield the cells from toxicity. Nevertheless, our findings underscore the suitability of PEI-CD as a biocompatible coating, paving the way for its application in various biochemical and catalytic processes.

### Protective effects

**Fig. 3:**
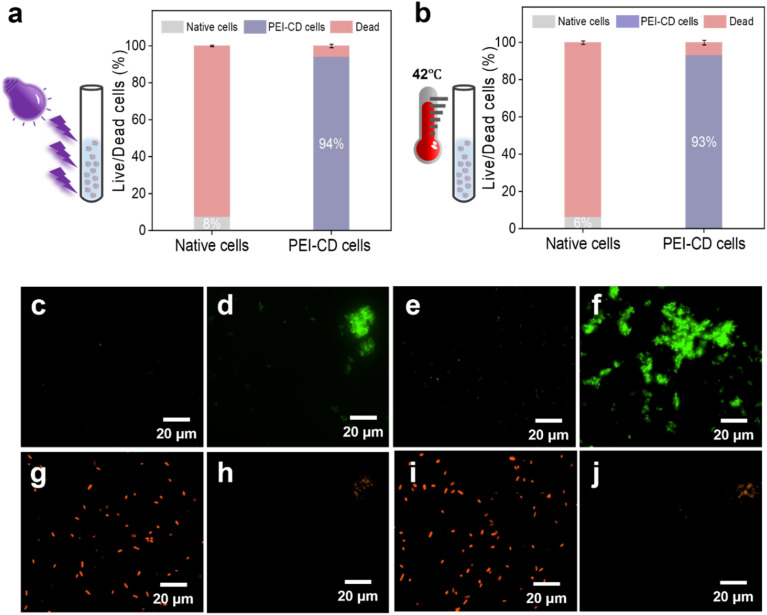
Effect of PEI-CD coating on cell viability under harsh environmental conditions. Cell viability of native and PEI-CD-coated cells after exposure to: (a) 1 hour of UV irradiation and (b) high temperature. Fluorescence microscopy images of native cells (c and g) and PEI-CD-coated cells (d and h) following UV radiation exposure. Fluorescence microscopy images of native cells (e and i) and PEI-CD-coated cells (f and j) following high-temperature exposure. The results represent average values derived from three parallel experiments. Error bars correspond to the standard deviations from these three measurements (*n* = 3).

Building on the promising cell viability results, we investigated whether PEI-CD could protect cells from various external stressful conditions (Fig. 3). To evaluate the effect of each stress condition separately, native cells were used as untreated control samples.

First, we assessed cell viability after exposure to UV radiation. After 1 hour of UV irradiation, the majority of native cells were dead, as confirmed by the live/dead assay (Fig. 3a, c and g). In stark contrast, approximately 97% of PEI-CD-coated cells survived UV exposure (Fig. 3d and h). A similar protective effect was observed under high-temperature stress at 42 °C (Fig. 3b, e, f, i and j). After 1 hour of exposure, only 9% of native cells remained alive (Fig. 3e and i), whereas 92% of PEI-CD-coated cells survived this treatment (Fig. 3f and j). This suggests that the PEI-CD polymer physically shields cells from damage induced by UV radiation and high temperatures. Moreover, PEI-CD-coated cells demonstrated higher tolerance to interfacial stress (water-organic two-phase system) compared to native cells, as shown by fluorescence microscopy (Fig. S9 and S10†). Approximately 94% of native cells died under interfacial stress, while 95% of PEI-CD-coated cells survived similar conditions. The coating also provided significant protection against 5% acetonitrile exposure (Fig. S11 and S12†).

These findings indicate that PEI-CD coating significantly enhances cellular integrity and viability under various environmental stressors, thereby expanding its potential applications under harsh industrial conditions.

**Fig. 4:**
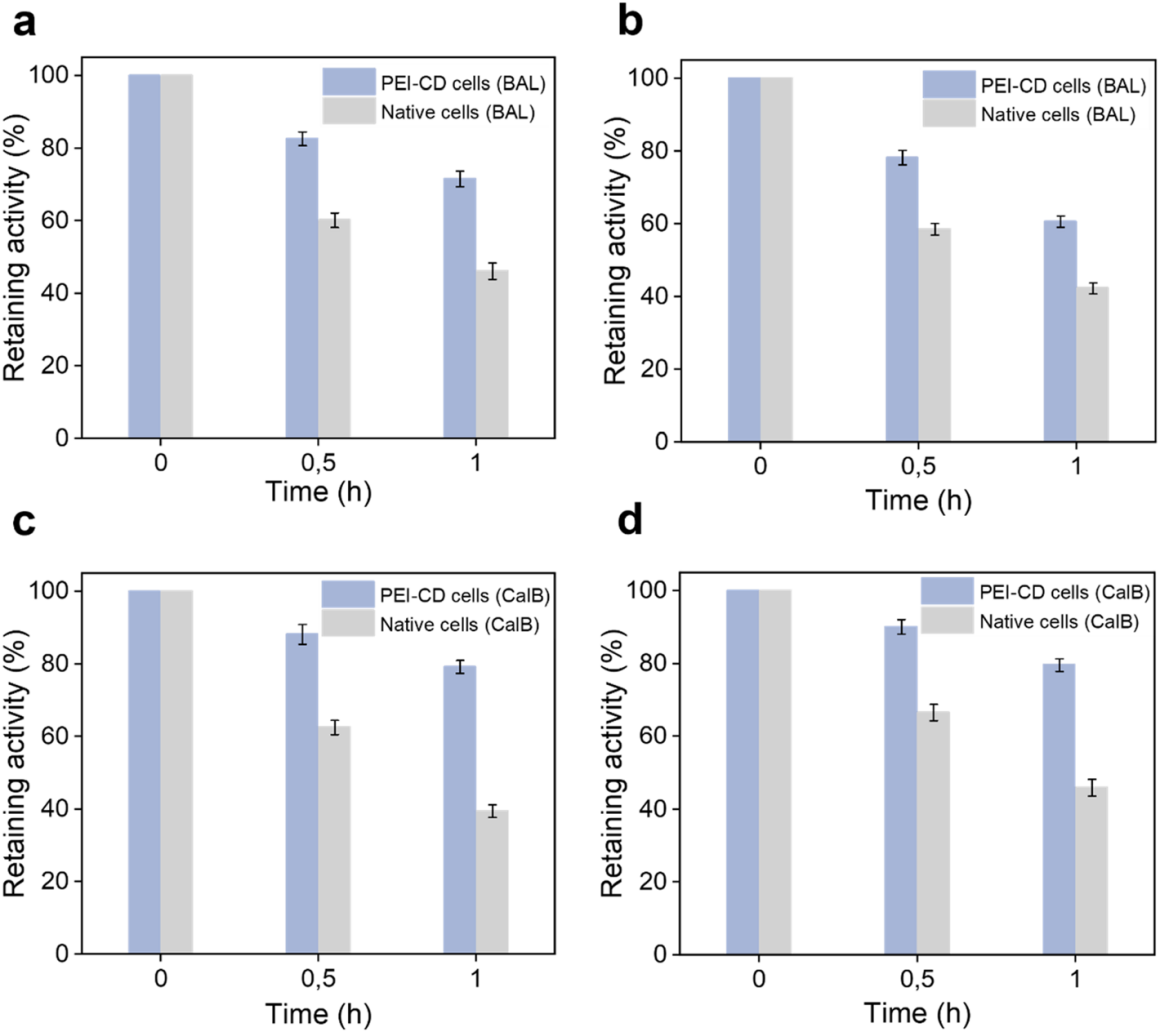
Enzyme activity in PEI-CD cells exposed to harsh environmental conditions. BAL activity after exposure to (a) high temperature or (b) UV irradiation. CalB activity after exposure to (c) high temperature and (d) UV irradiation. The above results represent the average values derived from three parallel experiments. The error bars depicted in the figures correspond to the standard deviations obtained from these three parallel measurements, denoted as *n* = 3.

In addition to cell viability, we were interested in whether PEI-CD coating can protect intracellular enzymes. To investigate this, two distinct enzymes, benzaldehyde lyase (BAL) and *Candida antarctica* lipase B (CalB), were independently overexpressed in *E. coli* cells and then treated with various external harsh conditions. Interestingly, as shown in Fig. 4a, the relative BAL activity in native cells decreased to 46% after exposure to high temperatures, whereas in PEI-CD-coated cells, it remained at approximately 71%. Similarly, after 1 hour of UV irradiation, BAL activity in PEI-CD-coated cells was significantly higher (60%) compared to native cells (42%) (Fig. 4b). Comparable protective effects were observed for PEI-CD-coated cells under treatment with 5% acetonitrile and interfacial stress (Fig. S13 and S14†). Furthermore, the PEI-CD coating led to an improved CalB activity under similar conditions. For example, PEI-CD-coated cells retained 80% of their activity after exposure to high temperatures or UV irradiation, whereas native cells exhibited only about 40% activity (Fig. 4c and d). A similar improvement of retained CalB activity was also observed after treatment with 5% acetonitrile or after interfacial stress (Fig. S15 and S16†). Consequently, these results collectively demonstrate that PEI-CD coating provides protective effects to the intracellular enzymes, which is of great interest for their catalytic use.

### Catalytic performance in single-step reactions

**Fig. 5:**
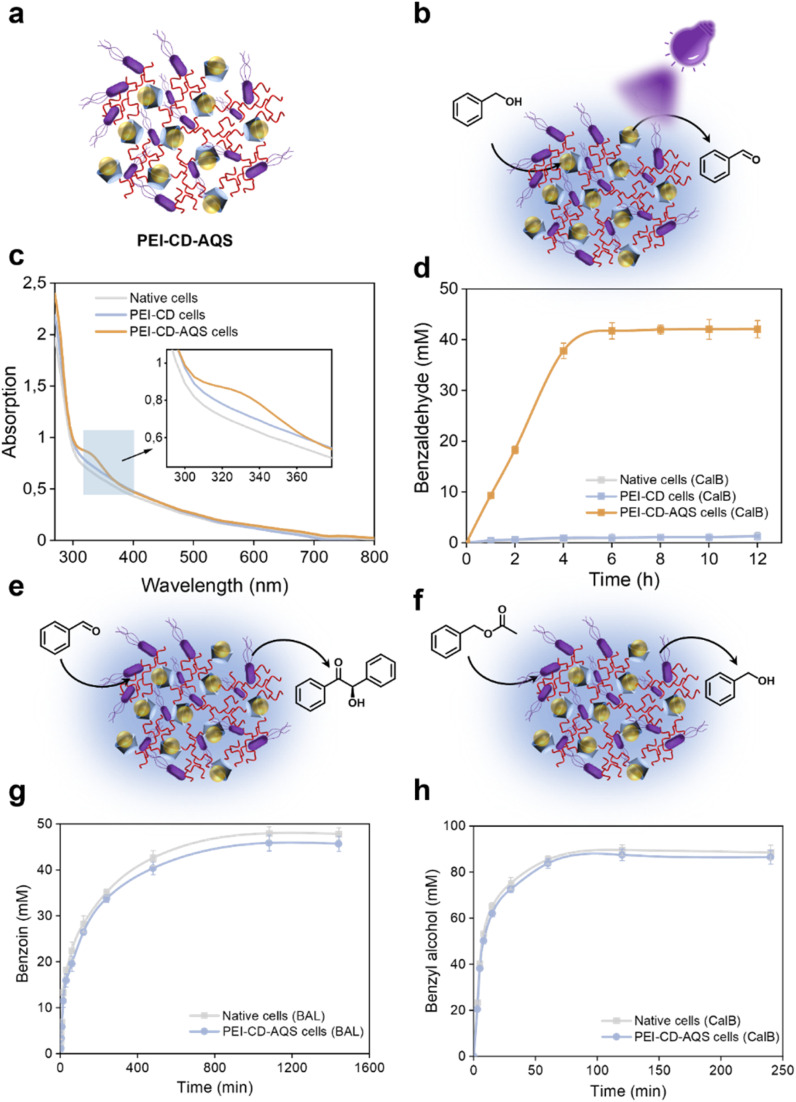
Catalytic performance of PEI-CD-AQS cells in single-step photochemical and enzymatic reactions. (a) Scheme of PEI-CD-AQS cells. (b) Benzyl alcohol oxidation by PEI-CD-AQS cells overexpressing CalB. (c) UV-vis spectra of native, PEI-CD-coated, and PEI-CD-AQS-coated *E. coli* cells. (d) Catalytic activity of CalB for benzyl alcohol oxidation in PEI-CD-AQS cells. (e) Benzoin condensation catalyzed by PEI-CD-AQS cells overexpressing BAL. (f) Benzyl acetate hydrolysis catalyzed by PEI-CD-AQS cells overexpressing CalB. (g) Catalytic activity of BAL for benzoin condensation in PEI-CD-AQS cells. (h) Catalytic activity of CalB for benzyl acetate hydrolysis in PEI-CD-AQS cells.

The remarkable impact of PEI-CD modification inspired us to use it as a way to incorporate photocatalysts within the cell surface, producing photoenzymatic systems. As shown in Fig. 5a, AQS was first complexed with PEI-CD. This was confirmed by UV-vis spectroscopy (Fig. 5c) through the appearance of a peak at 330 nm characteristic of AQS. The amount of AQS captured in the polymeric system was also quantified using a calibration curve (Fig. S17 and S18†). Table S1† illustrates that variation in the concentration of PEI-CD lead to corresponding variations in catalyst loading. Consequently, the PEI-CD-AQS system that presented the highest catalytic activity was used to photooxidize benzyl alcohol to benzaldehyde. The PEI-CD-AQS system exhibited excellent catalytic performance compared to that of native and PEI-CD cells (Fig. S19†), further confirming the feasibility of this inclusion strategy based on the host–guest chemistry.

The combination of chemo- and biocatalysts in a single chemoenzymatic system brings added value by improving efficient mass transfer and eliminating the need for intermediate product isolation and purification. Nevertheless, it is not uncommon for the combination of these two distinct catalytic systems to lead to mutual inactivation. To test these interactions in our system, we evaluated the oxidation of benzyl alcohol in PEI-CD-AQS cells overexpressing CalB (Fig. 5b). Benzyl alcohol was oxidized to benzaldehyde in the presence of PEI-CD-AQS and UV irradiation to a maximum concentration of 40 mM (Fig. 5d), which was similar to that obtained when combining a similar amount of free AQS with native cells. This confirms that the photocatalyst maintained excellent photocatalytic performance after complexation.

The activity of the model benzoin condensation reaction catalyzed by BAL was also evaluated (Fig. 5e). A final product concentration of over 45 mM was obtained in PEI-CD-AQS cells, which was similar to that of the control group (Fig. 5g). Likewise, the hydrolytic activity of CalB was also maintained in the presence of the photocatalyst (Fig. 5f and h). The concentration of produced benzaldehyde gradually increased with time until a plateau of 85 mM was reached at 120 min with no major differences between PEI-CD-AQS cells and control cells, indicating that the photocatalyst did not affect the enzyme activity. The PEI-CD-coated systems demonstrated good compatibility between photocatalyst and biocatalyst, which provides great potential for application in cascade reactions.

### Recyclable photobiocatalytic cascade synthesis

**Fig. 6:**
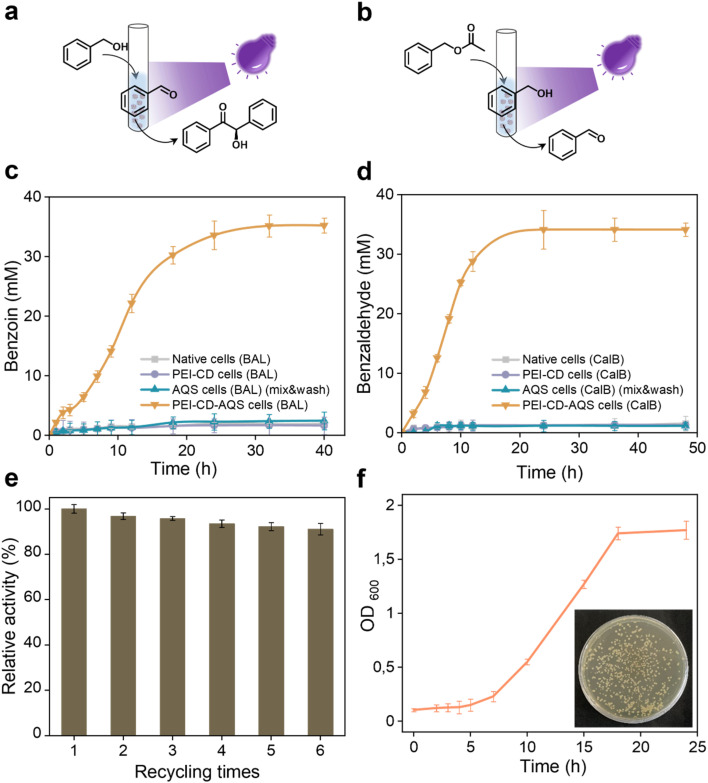
Catalytic performance of PEI-CD-AQS systems in cascade reactions. (a) One-pot cascade reaction using PEI-CD-AQS cells overexpressing BAL. (b) One-pot cascade reaction using PEI-CD-AQS cells overexpressing CalB. (c) Benzoin production over time in a one-pot cascade reaction. (d) Benzaldehyde production in a one-pot cascade reaction. (e) Activity of the PEI-CD-AQS cells (BAL) system after reusing it up to six times. (f) Growth curve and proliferation (inset) of cultured cells after recycling.

Given the compatibility of the aforementioned photocatalyst and whole-cell systems, we were encouraged to explore their combination in a one-pot cascade reaction. Using PEI-CD-AQS cells overexpressing benzaldehyde lyase (BAL), we aimed to achieve a two-step cascade synthesis: photo-oxidation of benzyl alcohol to benzaldehyde, followed by BAL-catalyzed condensation to (*R*)-benzoin (Fig. 6a). Although hydrogen peroxide, such as that generated during photooxidation by AQS, can significantly inactivate enzymes, this effect was not observed in our PEI-CD-AQS system overexpressing BAL. In fact, the cascade reaction using this system reached a final product concentration of over 35 mM (Fig. 6c), which is over 18 times higher than that of other control systems, which include native cells, PEI-CD-coated cells, and AQS-containing cells (after mixing and washing), all overexpressing BAL. This enhanced photo-biocatalytic conversion is likely due to the protective effects of the coated cell surface, shielding the cells from reactive oxygen species (ROS) generated during the reactions.

Expanding on this success, we investigated whether this photobiocatalytic system could be extended to other enzymes and cascade reactions. To this end, we combined AQS and CalB to perform another cascade reaction (Fig. 6b), where benzyl acetate was enzymatically converted to benzyl alcohol, followed by photooxidation to form benzaldehyde. As shown in Fig. 6d, PEI-CD-AQS cells overexpressing CalB achieved a final benzaldehyde concentration of 34 mM after 24 hours, which is over 28 times that of other control groups, which include native cells, PEI-CD-coated cells, and AQS-containing cells (after mixing and washing), all overexpressing CalB.

Most interestingly, the physical attachment of the photocatalyst AQS to the cell surface enabled its recycling through simple centrifugation and washing. This recyclability was demonstrated by reusing the PEI-CD-AQS system six times without significant loss in cascade performance (Fig. 6e). Furthermore, the recovered cells remained active and proliferative after recycling (Fig. 6f).

Therefore, PEI-CD-coated cells overexpressing enzymes can complex photocatalysts such as AQS, enabling efficient and recyclable one-pot photobiocatalytic synthesis.

## Conclusion

In conclusion, this study presents chemoenzymatic cascade reactions by introducing a supramolecular host–guest strategy for attaching photocatalysts to living bacterial cells. The approach of using β-cyclodextrin-functionalized polyethylenimine (PEI-CD) polymers to coat *E. coli* cells demonstrates exceptional biocompatibility and protective effects against harsh conditions such as UV radiation, high temperatures, and organic solvents. The successful integration of anthraquinone-2-sulfonic acid sodium salt (AQS) as a guest photocatalyst onto the cell surface highlights the potential of this method for efficient photobiocatalytic reactions. This system is comparable to other bacterial coatings recently reported, including cell surface polymerization^35^ and artificial sporulation,^36^ by granting significantly improved resistance to external factors and improved biocatalysis. This is likely achieved by providing a protective physical barrier around the cell, preventing damage by external stress, improving substrate diffusion, and shielding it against the action of harmful chemical agents such as singlet oxygen.^36^ Furthermore, it holds a competitive advantage over these by combining the catalytic versatility of choosing from different host–guest systems available and the simplicity of host–guest interactions. By reducing their synthetic complexity, it opens new avenues for the design of hybrid catalytic systems that combine chemical and biological catalysts in a plug-and-play manner. This concept can be extended to various green chemistry and advanced synthesis applications, offering a promising platform for sustainable and efficient chemical synthesis. Additionally, the reversible nature of host–guest interactions suggests potential applications in drug delivery and biosynthesis, warranting further exploration.

## Data availability

The data supporting this article have been included as part of the ESI.†

## Author contributions

J. Zhang designed and performed the experiments and wrote the manuscript. V. F. Batista designed and performed experiments and revised the manuscript. C. Wu conceived the study and was responsible for the project administration and manuscript revision. All authors discussed the results and commented on the manuscript. All authors contributed to this work and have approved the final version of the manuscript.

## Conflicts of interest

The authors declare they have no conflicts of interest.

## Supplementary Material

SC-OLF-D4SC06508E-s001
