# Supplementary material for: Host–guest chemistry on living cells enabling recyclable photobiocatalytic cascade
Source: Chem Sci. 2025 Feb 6;16(10):4480–9. doi: 10.1039/d4sc06508e (PMC11801380; doi:10.1039/d4sc06508e)
Supplement: SC-016-D4SC06508E-s001 [file SC-016-D4SC06508E-s001.pdf]

Supplementary Information (SI) for Chemical Science.
This journal is © The Royal Society of Chemistry 2025

Supporting information

Host-guest Chemistry on Living Cells Enabling Recyclable Photobiocatalytic Cascade

Table of Contents

1. Materials and instruments 3

2. Synthesis 4

2.1 Synthesis of PEI-CD 4

2.2 Synthesis of 1-Adamantanecarbonyl chloride 4

2.3 Synthesis of Compound 1 4

2.4 Synthesis of AdaFITC 5

3. Preparation of PEI-CD cells 5

4. Preparation of PEI-CD-AdaFITC cells 6

5. Preparation of PEI-CD-AQS cells 6

6. Enzyme Expression 6

6.1 Expression of benzaldehyde lyase (BAL) 6

6.2 Expression of *Candida antarctica* lipase B (CalB) 6

7. Sample preparation for electron microscopy 7

8. Cell plating assay 8

9. Cell viability assay 8

10. Re-culturing and cell growth curve 8

11. Catalytic performance 8

11.1 Whole-cell catalysis with PEI-CD-coated cells overexpressing BAL 9

11.2 Whole-cell catalysis with PEI-CD-coated cells overexpressing CalB 9

11.3 Chemoenzymatic cascade with AQS and BAL 9

11.4 Chemoenzymatic cascade reaction with AQS and CalB 10

12. Recycling of PEI-CD-AQS cells (CalB/BAL) for cascade reaction 11

13. Supplementary results 12

14. References 31

1. Materials and instruments

All reagents were used without further purification unless mentioned. β-Cyclodextrin (β-CD), branched polyethylenimine (PEI) with an average molecular weight of 600 Da, 1,1′-carbonyldiimidazole (CDI), 1-Adamantanecarboxylic acid, 2,2′-(ethylenedioxy)bis(ethylamine) (2,2'-EDBEA), oxalyl chloride, potassium carbonate, hydrochloric acid, fluorescein isothiocyanate, and anthraquinone-2-sulfonic acid sodium salt monohydrate (AQS) were purchased from Sigma Aldrich. Propidium iodide and SYTO^{TM} 9 were purchased from Thermo Fisher Scientific.

^{1}H NMR spectra were recorded on a Bruker AVANCE III 400Hz spectrometer equipped with tunable multinuclear probes and an auto-sampler using 5-mm NMR tubes at 25 ºC. UV-Vis spectra were recorded on a Cary 5000 UV-Vis-NIR spectrophotometer (Agilent Technologies) at room temperature using a 10-mm quartz cell. Thermogravimetric analysis (TGA) was done with a TG 50 modular unit and a TC 15 TA controller (Mettler-Toledo, Giessen, Germany) by increasing the temperature under a nitrogen atmosphere from 35 to 800 °C at 10 K/min, holding for 5 min at 800 °C, and cooling from 800 °C to 35 °C at 100 K/min. Scanning electron microscopy (SEM) was performed using a S-4800 microscope (Hitachi) operated at an accelerating voltage of 1 kV. Transmission electron microscopy (TEM) was performed on a Jeol JEM 1400 Plus instrument equipped with a Jeol Ruby camera and the Picture Overlay Program for correlative light electron microscopy (CLEM).

Epifluorescent images of bacteria were acquired using a Nikon Ti2 widefield microscope with NIS-Elements AR 5.30 software and a 100x/1.45 NA oil objective. Channels were set up in the 488 nm (FITC) and 561 nm (TRITC) channel using a CoolLED “pE-300 white” light source, standard FITC and TRITC filter cubes, and an Andor Zyla monochrome camera. The contrast was adjusted to the desired levels in ImageJ ver. 1.53v. Gas Chromatography (GC) analysis was carried out on a Shimadzu Nexis^{TM} GC-2030 gas chromatograph equipped with an AOC-20i/s autoinjector/autosampler.

2. Synthesis

2.1 Synthesis of PEI-CD

The procedure for the synthesis of PEI-CD polymer followed the method described in the literature.[1] Briefly, the solid reagents were dried in a glass oven at 50 °C overnight before use. Then, β-CD (1.12 g, 1 mmol) was dissolved in 20 mL of anhydrous DMSO, followed by the addition of 1,1′-carbonyldiimidazole (CDI, 1.30 g, 8 mmol). The solution was stirred at room temperature for 8 h. Next, 30 mL of PEI (4.32 g, 7.20 mmol) dissolved in anhydrous DMSO were added dropwise with stirring. The reaction mixture was stirred for additional 12 h. The resulting PEI-β-cyclodextrin conjugate was extensively dialyzed against double-distilled water using a dialysis membrane with a molecular weight cut off (MWCO) of 2000 Da to remove any impurities prior to lyophilization to dryness.

2.2 Synthesis of 1-Adamantanecarbonyl chloride

1-Adamantanecarbonyl chloride was synthesized according to the literature.[2] 1-Adamantanecarboxylic acid (4 g, 22 mmol) was dissolved in 100 mL dichloromethane, and a catalytic amount of DMF was added. Before dropwise addition of oxalyl chloride (1.5 mL, 18 mmol), the mixture was cooled to 0°C. The reaction was then allowed to warm to room temperature and stirred for additional three hours. Completion of the reaction was verified by TLC, and the solvent was removed under reduced pressure to isolate adamantanecarbonyl chloride. The white solid was used without further purification.

2.3 Synthesis of Compound 1

After vacuum drying with heating for 30 minutes, 1.2 g of potassium carbonate was added to 40 mL of anhydrous DCM along with 3 mL (20 mmol) of 2,2′-(ethylenedioxy)bis(ethylamine) (2,2'-EDBEA). The reaction vessel was sealed and cooled to 0 °C in an ice bath. In a separate container,

adamantanecarbonyl chloride (2 g, 10 mmol) was dissolved in anhydrous DCM (20 mL) and cooled to 0°C before being added dropwise to the first mixture. After addition, the reaction mixture was allowed to warm to room temperature and stirred for additional 2 hours, where TLC analysis confirmed that the reaction was complete. The mixture was then filtered to remove excess potassium carbonate, washed once with water (20 mL), and then acidified with hydrochloric acid (10% w/v, 4 mL) before extracting the organic phase with water (3 × 30 mL). The combined aqueous layers were washed with DCM (3 × 20 mL) before adjusting the pH to 8 with NaOH (10% w/v). Compound 1 was then extracted with DCM (5 × 30 mL), and the combined organic layers were washed with brine and dried using anhydrous sodium sulfate before being concentrated under reduced pressure. Compound 1 was obtained as a clear, viscous liquid.

2.4 Synthesis of AdaFITC

A solution of fluorescein isothiocyanate (FITC) (purity: 95 %, 1g, 2.43 mmol) in DMF (10.0 mL) was treated with compound 1 (940 mg, 3.0 mmol) and stirred for 2 hours. The solvent was evaporated, and the residue was purified by flash chromatography (ethyl acetate/methanol 9:1) to give AdaFITC (1.46 g, 4.7 mmol, 82%) as a light-yellow viscous liquid. ESI-MS m/z: $[M + H]^+$ Calculated for $C_{38}H_{42}N_3O_8S$: 700.27; Found: 700.2.

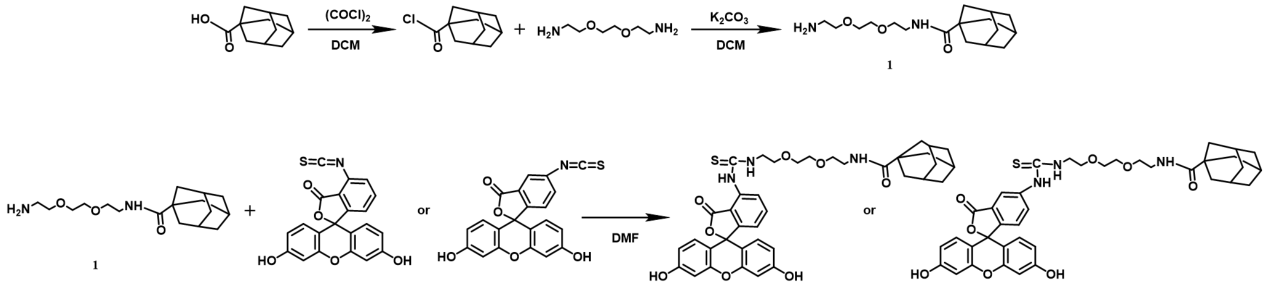

Scheme 1. Synthesis of AdaFITC

3. Preparation of PEI-CD cells

80 mg of PEI-CD dissolved in KPi buffer were combined with *E. coli* cells re-suspended in 1 mL KPi buffer to reach a final OD_{600} of 2.0. This mixture was stirred at 4 ºC for 30 minutes. After centrifugation, the cells were washed with KPi buffer 3 times before being stored.

4. Preparation of PEI-CD-AdaFITC cells

80 mg of PEI-CD dissolved in KPi buffer and *E.coli* cells re-suspended in 1 mL KPi buffer were combined to reach a final OD_{600} of 2.0. The above mixture was stirred at 4 ºC for 30 minutes. After centrifugation, the cells were washed with KPi buffer 3 times. Afterward, the cells were re-suspended in 1 mL KPi buffer, followed by adding AdaFITC dissolved in DMSO dropwise. Finally, the resulting suspension was centrifuged for 30 minutes, and the cells were washed with a KPi/10% DMSO solution.

5. Preparation of PEI-CD-AQS cells

80 mg of PEI-CD dissolved in KPi buffer and *E. coli* cells re-suspended in 1 mL KPi buffer were combined to reach a final OD_{600} of 2.0. The above mixture was stirred at 4 ºC for 30 minutes. After centrifugation, the cells were washed with KPi buffer 3 times. Afterward, the cells were re-suspended in 1 mL KPi buffer, followed by dropwise addition of an aqueous solution of AQS. The cells were centrifuged after 30 minutes and washed with KPi buffer.

6. Enzyme Expression

6.1 Expression of benzaldehyde lyase (BAL)

BAL was expressed in the recombinant strain *E. coli* BL21(DE3) containing a pET28a plasmid. The expression procedure is similar to that previously reported[3]. *E. coli* cells were incubated in lysogeny broth (LB)/kanamycin medium overnight at 37 °C. The preculture was then transferred to the main culture and incubated at 37 °C until reaching an OD_{600} of 0.6, whereby a final concentration of 0.8 mM isopropyl β-D-1-thiogalactopyranoside (IPTG) was added to induce expression. After that, the cells were further incubated for 16 h and finally collected by centrifugation (4 ^{o}C, 5000 rpm, 12 min).

6.2 Expression of *Candida antarctica* lipase B (CalB)

CalB was expressed according to the following protocol[4]: *E. coli* BL21(DE3) cells containing the pET22b_CalB-His plasmid (after expressed CalB is localized in periplasm) were incubated at 37 °C

in LB medium containing 100 µg/mL ampicillin overnight. This preculture was used to inoculate a main culture with autoinduction medium containing 100 µg/mL ampicillin. The main culture was incubated at 20 °C overnight.

Auto-Induction Medium (2L) consists of 20 g Tryptone, 10 g Yeast extract, 40 mL 50xM stock solution, 4 mL 1M $MgSO_4$, 0.4 mL 1000x Trace Metals stock solution and 40 mL 50x 5052 stock solution. Add several sterilized stocks to autoclaved Tryptone and Yeast medium right before use. The stock solutions include 50x salt stock solution (1.25 M Na_2HPO_4x7 H_2O, 1.25 M KH_2PO_4, 2.5 M NH_4Cl, 0.25M Na_2SO_4), 50x 5052 stock solution (25% Glycerol, 2.5% Glucose, 10% G-Lactose) and 1000x stock solution of trace metals (50 mM $FeCl_3$, 20 mM $CaCl_2$(x2 H_2O), 10 mM $MnCl_2$(x4 H_2O), 10 mM $ZnSO_4$(x7 H_2O), 2 mM $CoCl_2$(x6 H_2O), 2 mM $CuCl_2$, 2 mM $NiCl_2$, 2 mM Na_2MoO_4, 2 mM Ma_2SeO_3, 2 mM H_3BO_3).

7. Sample preparation for electron microscopy

Native and PEI-CD *E. coli* cells were dispersed in PBS buffer (100 mM, pH 7.4) containing 2% glutaraldehyde for primary fixation at 4 °C overnight. Then, the cells were centrifugated and washed three times with Mili-Q water. The cells were further treated with osmium tetroxide (1% in Mili-Q water) for post-fixation at 4 °C for 2 h. Next, the cells were washed with Mili-Q water three times. After that, the cells were dehydrated by a series of ethanol-in-water solutions for 15 min, changing the volumetric ratio from 30 to 50, 70, 75, 90, 95, and 100%. After dehydration, the cells were collected by centrifugation and used in next sample preparation steps for SEM and TEM analysis.

The dehydrated cells for SEM were placed onto a piece of Si wafer, and then imaged without further coating.

The dehydrated cells for TEM were first dispersed in three different mixture solutions containing propene oxide and embedding medium, in which the embedding medium content (v/v) was stepwise increased from 2:1 to 1:1 and 1:2, and the cells were respectively incubated in each mixture for 1 h. Subsequently, these treated cells were transferred into 100% embedding medium

and kept at 60 °C for polymerization for 24 h. The cells embedded in the resin were finally subjected to ultrathin cutting and stained with uranyl acetate for TEM analysis.

8. Cell plating assay

Typically, 1 mL of PBS buffer (pH 7.4) was used to suspend *E. coli* cells (native and PEI-CD cells) until a final OD_{600} of 0.2 was reached. A sterile loop was dipped into this diluted solution and used to smear an LB agar plate. The plates were then incubated at 30 °C for 48 h before counting the colonies and imaging.

9. Cell viability assay

First, PBS buffer (pH 7.4) was used to suspend native and PEI-CD cells. Then, SYTO™ 9 was added, the cells incubated for 15 min and were washed with PBS buffer (pH 7.4) three times. Next, propidium iodide was added to the suspension, followed by 15 min incubation. The free dye was removed by washing with PBS buffer (pH 7.4) three times. The cells were subjected to fluorescence microscopy after three rounds of washing in PBS buffer (pH 7.4).

10. Re-culturing and cell growth curve

To achieve a final OD_{600} of 0.1, native and PEI-CD cells were suspended in 1 mL of LB medium with 100 µg/mL ampicillin. Re-culturing was performed at 37 °C in a shaking incubator. To obtain the cell growth curve, the OD_{600} was measured using UV-Vis spectroscopy at set timepoints. The experiments were conducted in triplicate.

11. Catalytic performance

Enzyme activity (U) was measured as millimolar product generated per minute ($U = mM_{product}\ min^{-1}$). The enzymatic activity of BAL was determined by suspending the cells in 1 mL KPi buffer until a final OD_{600} of 2.0 was reached. Then, the reaction was started by adding 10.1 µL of benzaldehyde. Following a 10-minute room temperature mixing period, 100 µL ethyl acetate were used to extract a 10 µL sample of the reaction mixture, and then dried by anhydrous magnesium sulfate. After

centrifugation, 100 μL of the supernatant were extracted for GC analysis. The experiment was performed in triplicate.

11.1 Whole-cell catalysis with PEI-CD-coated cells overexpressing BAL

The enzymatic activity of BAL in PEI-CD cells was determined by suspending the cells in 1 mL KPi buffer to a final OD_{600} of 2.0. Afterwards, 10.1 μL of benzyaldehyde were introduced to initiate the benzoin condensation reaction. At every time interval, 100 μL ethyl acetate were used to extract a 10 μL sample of the reaction mixture, followed by drying with anhydrous magnesium sulfate. After centrifugation, 100 μL of the supernatant was taken for GC analysis. The experiment was performed in triplicate.

11.2 Whole-cell catalysis with PEI-CD-coated cells overexpressing CalB

The enzymatic activity of CalB in PEI-CD cells was determined by suspending the cells in 1 mL PBS buffer to a final OD_{600} of 2.0. Afterwards, 14.2 μL of benzyl acetate were introduced to initiate the hydrolysis reaction. At every time interval, 10 μL of reaction mixture were taken and extracted with 100 μL ethyl acetate, followed by drying with anhydrous magnesium sulfate. After centrifugation, 100 μL of the supernatant was taken for GC analysis. The experiment was performed in triplicate.

11.3 Chemoenzymatic cascade with AQS and BAL

The chemoenzymatic activity of BAL and AQS was determined by suspending the PEI-CD-AQS cells overexpressing BAL in 1 mL KPi buffer to a final OD_{600} of 2.0. Then, 10.3 μL benzyl alcohol were added to initiate the cascade reaction under UV light irradiation. At each time interval, 10 μL of reaction mixture were taken and extracted with 100 μL ethyl acetate, followed by drying with anhydrous magnesium sulfate. After centrifugation, 100 μL of the supernatant was taken for GC analysis. The experiment was performed in triplicate.

For the chemoenzymatic activity of the native cells overexpressing BAL, the native cells were suspended in 1 mL KPi buffer to a final OD_{600} of 2.0. Then, 10.3 μL benzyl alcohol were added to initiate the reaction under UV light irradiation. At each time interval, 10 μL of reaction mixture were taken and extracted with 100 μL ethyl acetate, followed by drying with anhydrous magnesium sulfate. After centrifugation, 100 μL of the supernatant was taken for GC analysis. The experiment was performed in triplicate.

For the chemoenzymatic activity of the PEI-CD cells overexpressing BAL, the PEI-CD cells were suspended in 1 mL KPi buffer to a final OD_{600} of 2.0. Then, 10.3 μL benzyl alcohol were added to initiate the cascade reaction under UV light irradiation. At each time interval, 10 μL of reaction mixture were taken and extracted with 100 μL ethyl acetate, followed by drying with anhydrous magnesium sulfate. After centrifugation, 100 μL of the supernatant was taken for GC analysis. The experiment was performed in triplicate.

For the chemoenzymatic activity of control group (AQS with cells overexpressing BAL), the cells were suspended in 1 mL KPi buffer to a final OD_{600} of 2.0. After mixing with AQS for 10 minutes, they were washed with KPi buffer three times. The cells were then resuspended in 1 mL KPi buffer to a final OD_{600} of 2.0. Then, 10.3 μL of benzyl alcohol were added to initiate the cascade reaction under UV light irradiation. At each time interval, 100 μL ethyl acetate were used to extract a 10 μL sample of reaction mixture, followed by drying with anhydrous magnesium sulfate. After centrifugation, 100 μL of the supernatant was taken for GC analysis. The experiment was performed in triplicate.

11.4 Chemoenzymatic cascade reaction with AQS and CalB

The chemoenzymatic activity of CalB and AQS was determined by suspending PEI-CD-AQS cells overexpressing CalB in 1 mL PBS buffer to a final OD_{600} of 2.0. Then, 14.2 μL benzyl acetate was added to initiate the cascade reaction under UV light irradiation. At each time interval, 100 μL ethyl acetate was used to extract a 10 μL sample of the reaction mixture, followed by drying with

anhydrous magnesium sulfate. After centrifugation, 100 μL of supernatant was taken for GC analysis. The experiment was performed in triplicate.

For the chemoenzymatic activity of native cells overexpressing CalB, the native cells were suspended in 1 mL PBS buffer to a final OD_{600} of 2.0. Then, 14.2 μL benzyl acetate was added to initiate the cascade reaction under UV light irradiation. At each time interval, 100 μL ethyl acetate was used to extract a 10 μL sample of the reaction mixture, followed by drying with anhydrous magnesium sulfate. After centrifugation, 100 μL of supernatant was taken for GC analysis. The experiment was performed in triplicate.

For the chemoenzymatic activity of control group (PEI-CD cells overexpressing CalB), the PEI-CD cells were suspended in 1 mL PBS buffer to a final OD_{600} of 2.0. Then, 14.2 μL benzyl acetate was added to initiate the cascade reaction under UV light irradiation. At each time interval, 100 μL ethyl acetate was used to extract a 10 uL sample of the mixture, followed by drying with anhydrous magnesium sulfate. After centrifugation, 100 μL of supernatant was taken for GC analysis. The experiment was performed in triplicate.

For the chemoenzymatic activity of AQS with cells overexpressing CalB, the cells were suspended in 1 mL PBS buffer to a final OD_{600} of 2.0. After mixing with AQS for 10 minutes, they were washed with PBS buffer three times. The cells were then resuspended in 1 mL PBS buffer to a final OD_{600} of 2.0. To initiate the cascade reaction, 14.2 μL of benzyl acetate was added under UV light irradiation. At each time interval, 100 μL ethyl acetate was used to extract a 10 μL sample of the reaction mixture, followed by drying with anhydrous magnesium sulfate. After centrifugation, 100 μL of supernatant was taken for GC analysis. The experiment was performed in triplicate.

12. Recycling of PEI-CD-AQS cells (CalB/BAL) for cascade reaction

After performing the cascade reaction, the PEI-CD-AQS cells (CalB/BAL) were collected by gentle centrifugation at 4000 rpm for 3 min and washed with KPi buffer (100 mM, pH 8). The supernatant

was extracted with ethyl acetate for GC analysis, and the collected cells were reused under similar cascade reaction conditions. All tests were carried out in triplicate.

13. Supplementary results

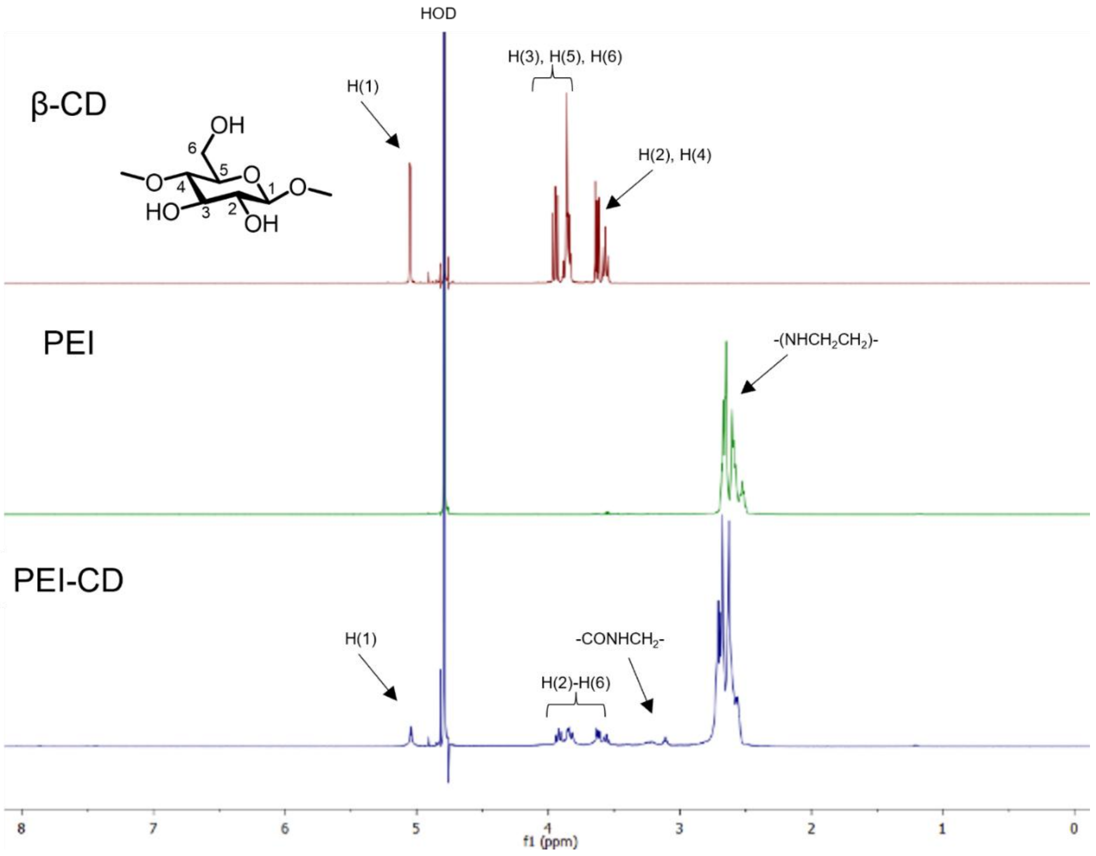

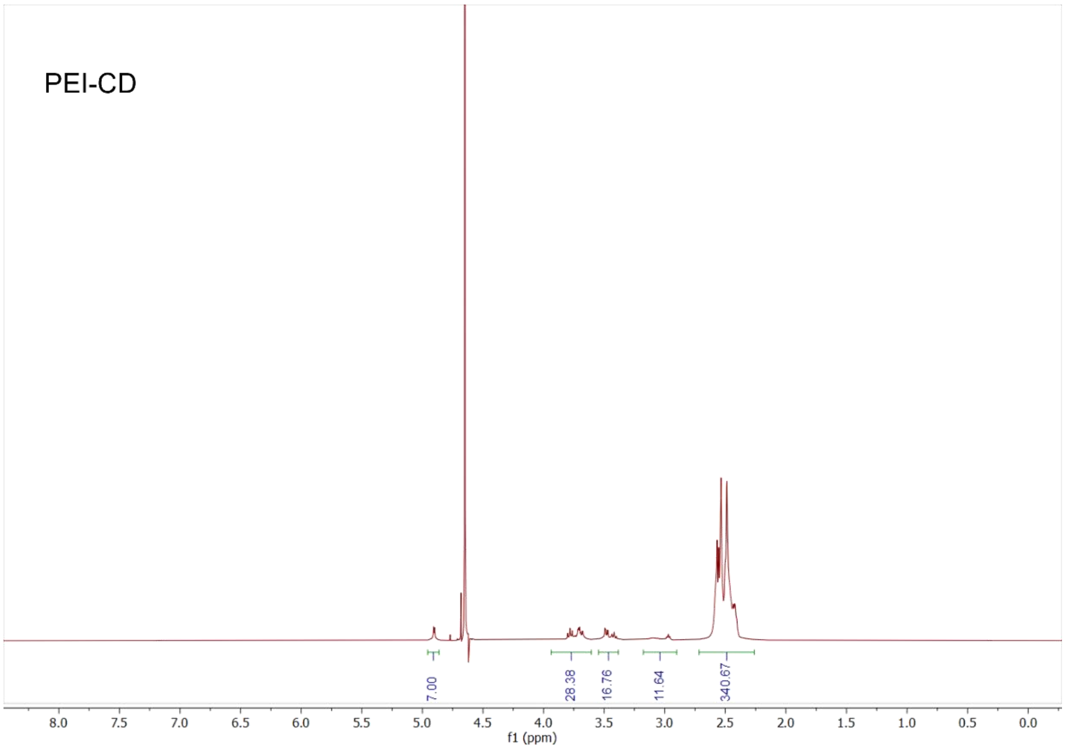

Figure S1. ^{1}H NMR spectra of β-CD, PEI, and PEI-CD.

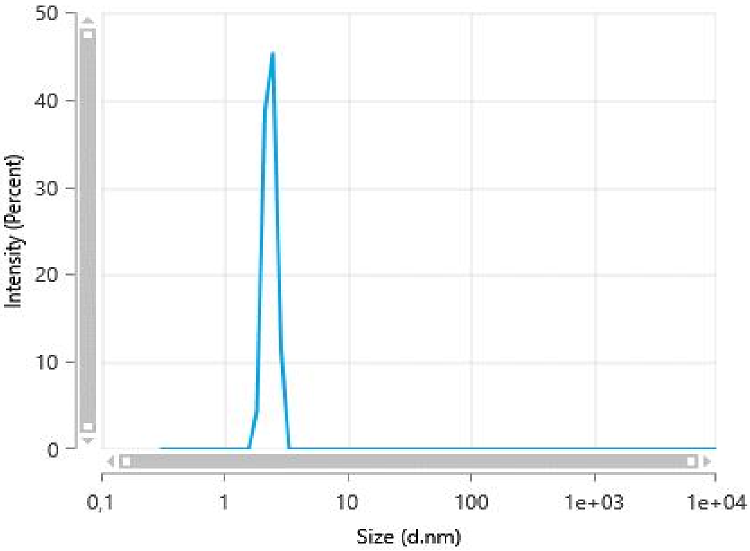

Figure S2. Dynamic Light Scattering spectrum of PEI-CD.

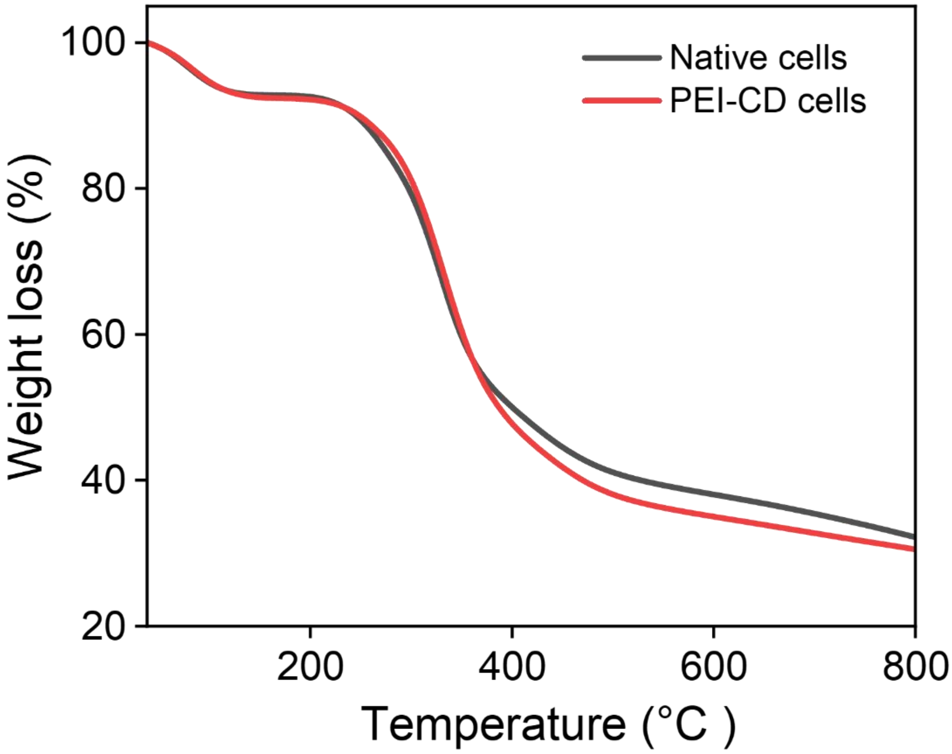

Figure S3. TGA of native (black) and PEI-CD (red) cells.

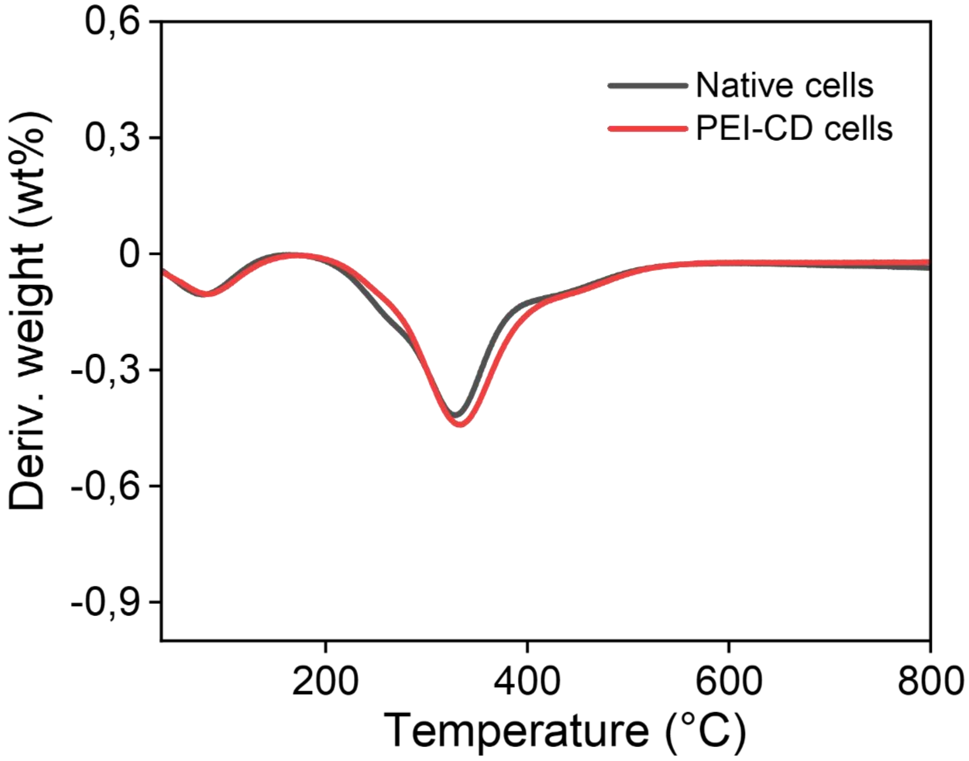

Figure S4. DTG of native (black) and PEI-CD (red) cells.

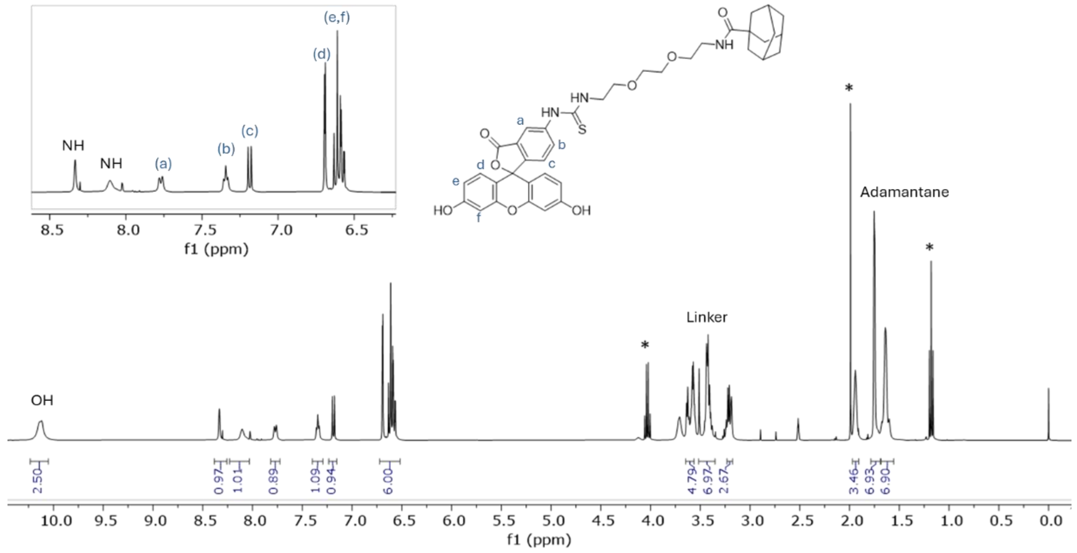

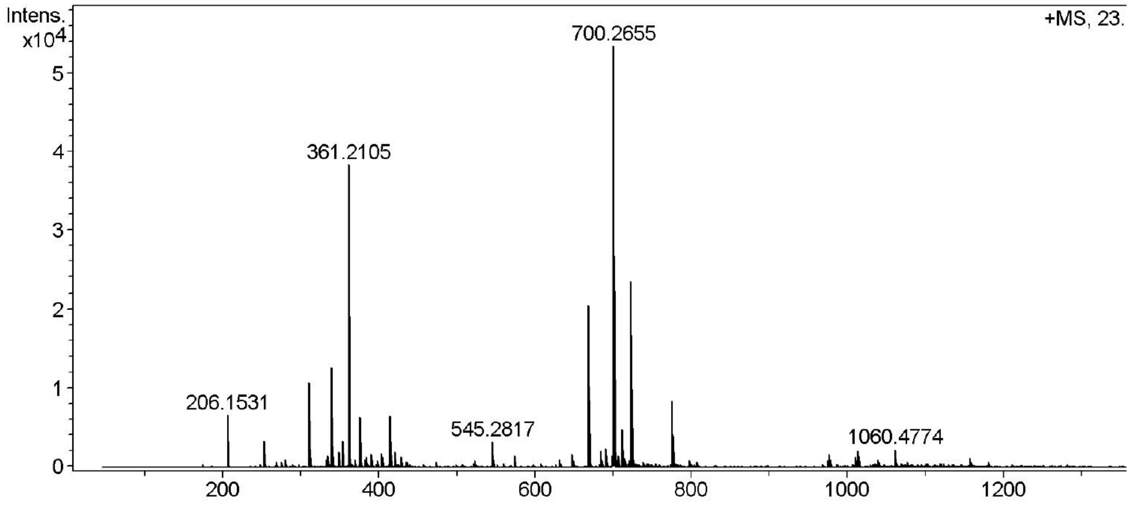

Figure S5. ^{1}H NMR and ESI-MS spectra of AdaFITC. *Residual solvents.

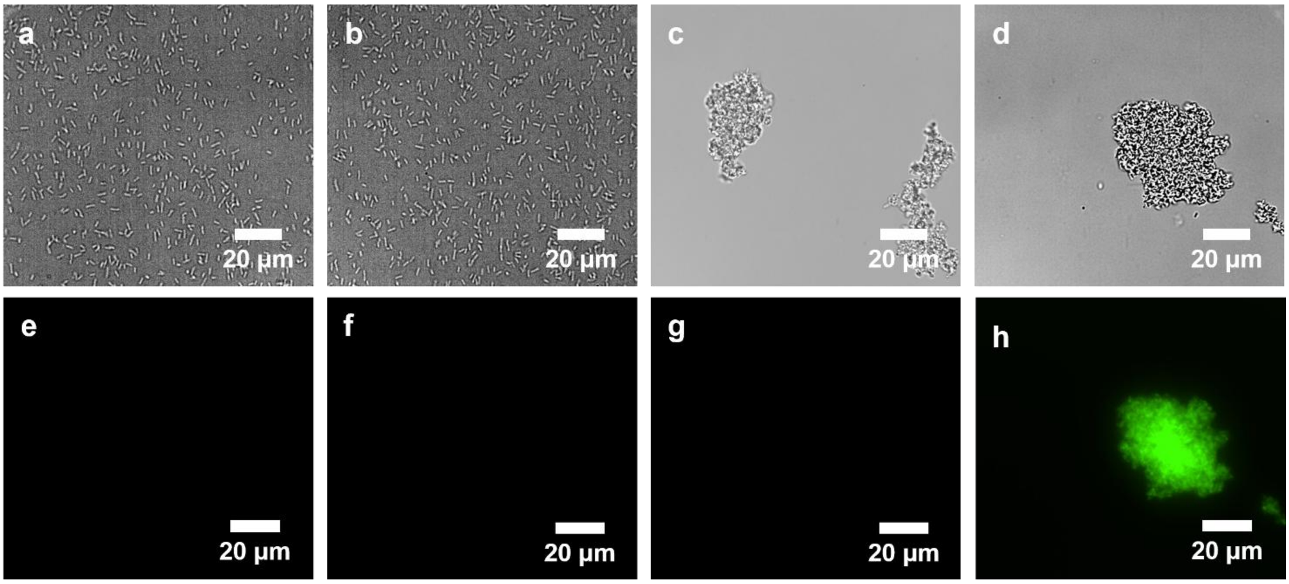

Figure S6. Bright-field and fluorescence microscopy images of native cells (a)(e), cells mixed with AdaFITC after a washing step (b)(f), PEI-CD cells (c)(g), and PEI-CD-AdaFITC cells (d)(h).

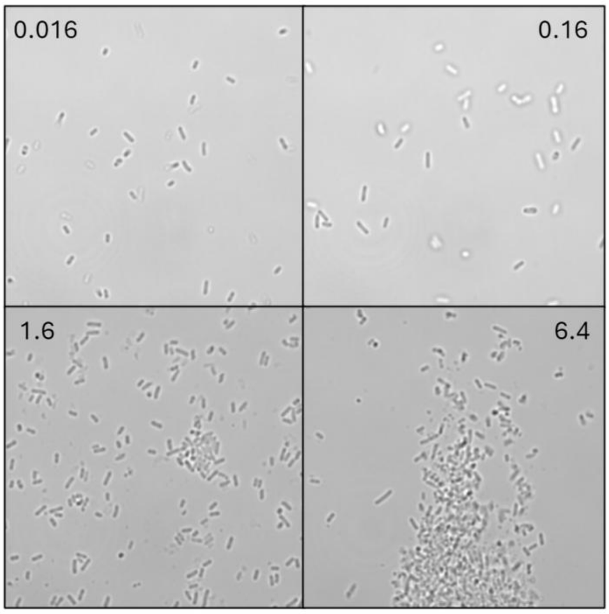

Figure S7. Bright-field microscopy images of *E. coli* cells after coating with different amounts of PEI-CD – 0.016 mg/mL, 0.16 mg/mL, 1.6 mg/mL, and 6.4 mg/mL.

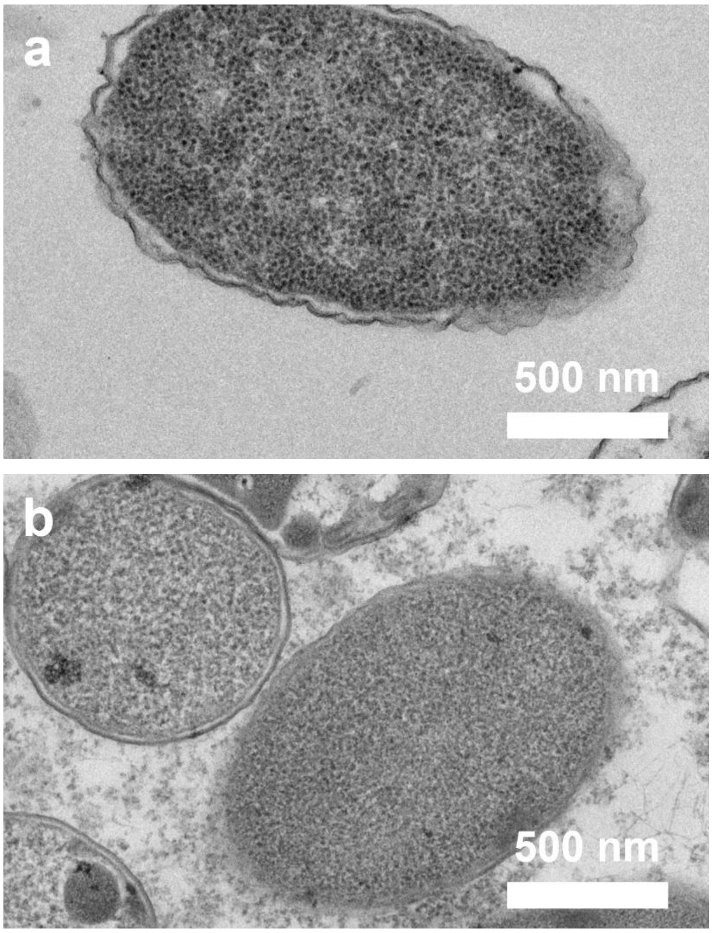

Figure S8. TEM images of (a) native and (b) PEI-CD cells.

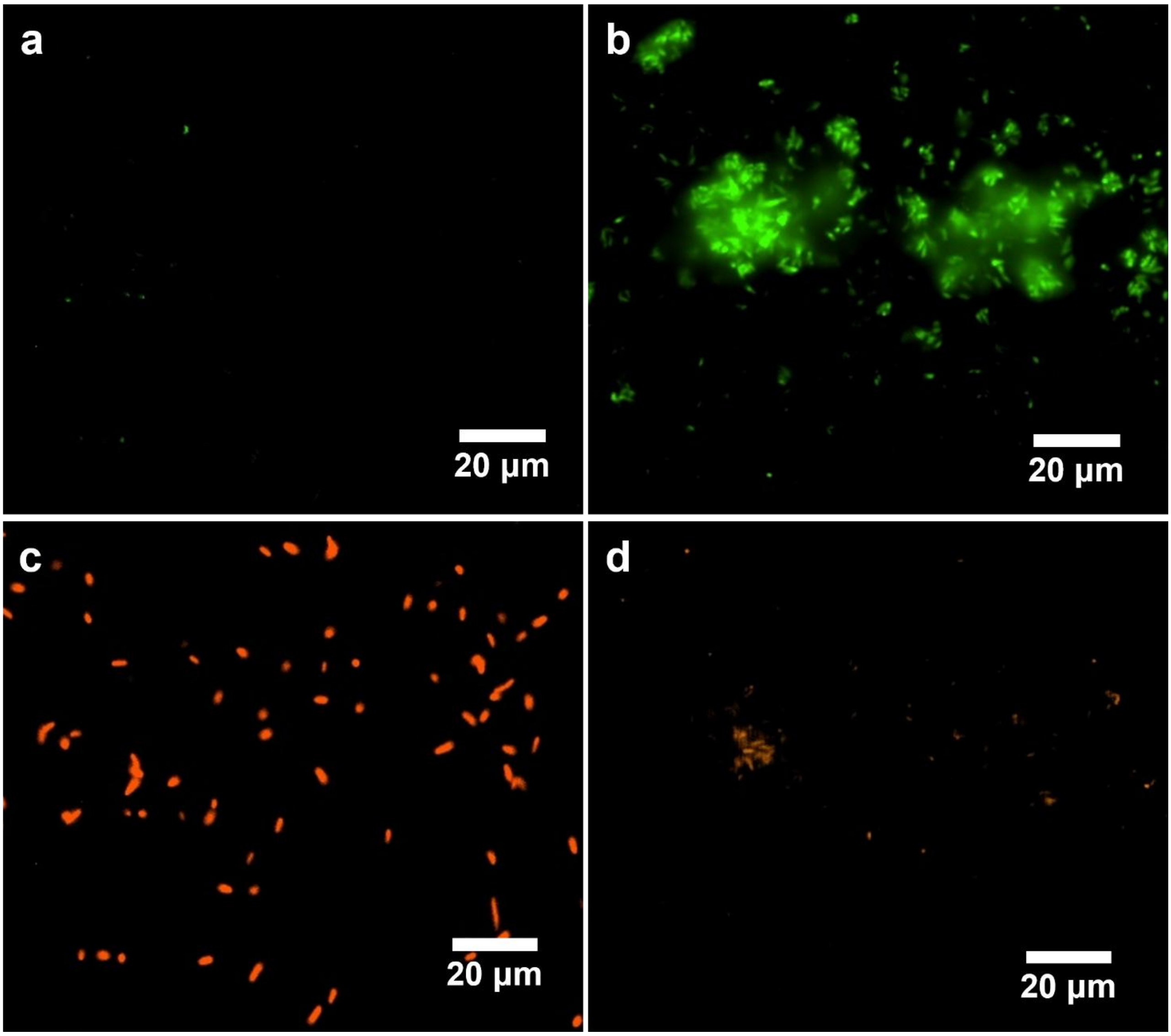

Figure S9. Live/Dead assay of native (a)(c) and PEI-CD (b)(d) cells after interfacial stress.

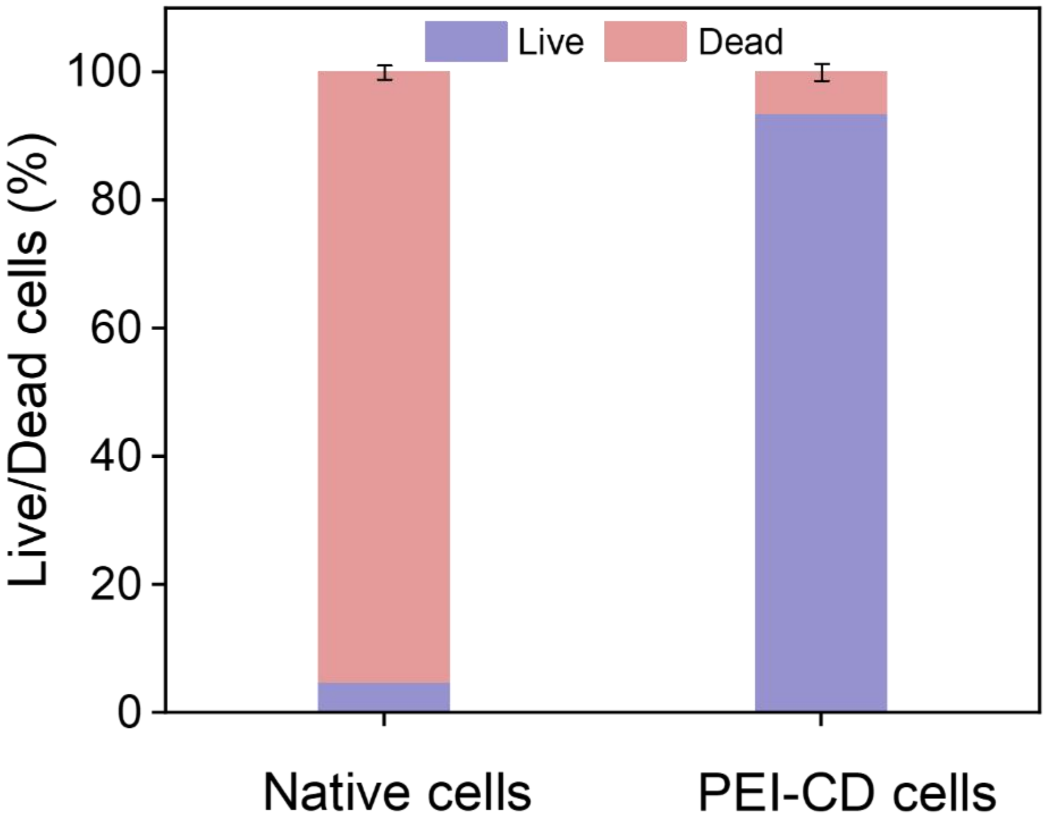

Figure S10. Quantification of live and dead cells from the microscopy assay in S7.

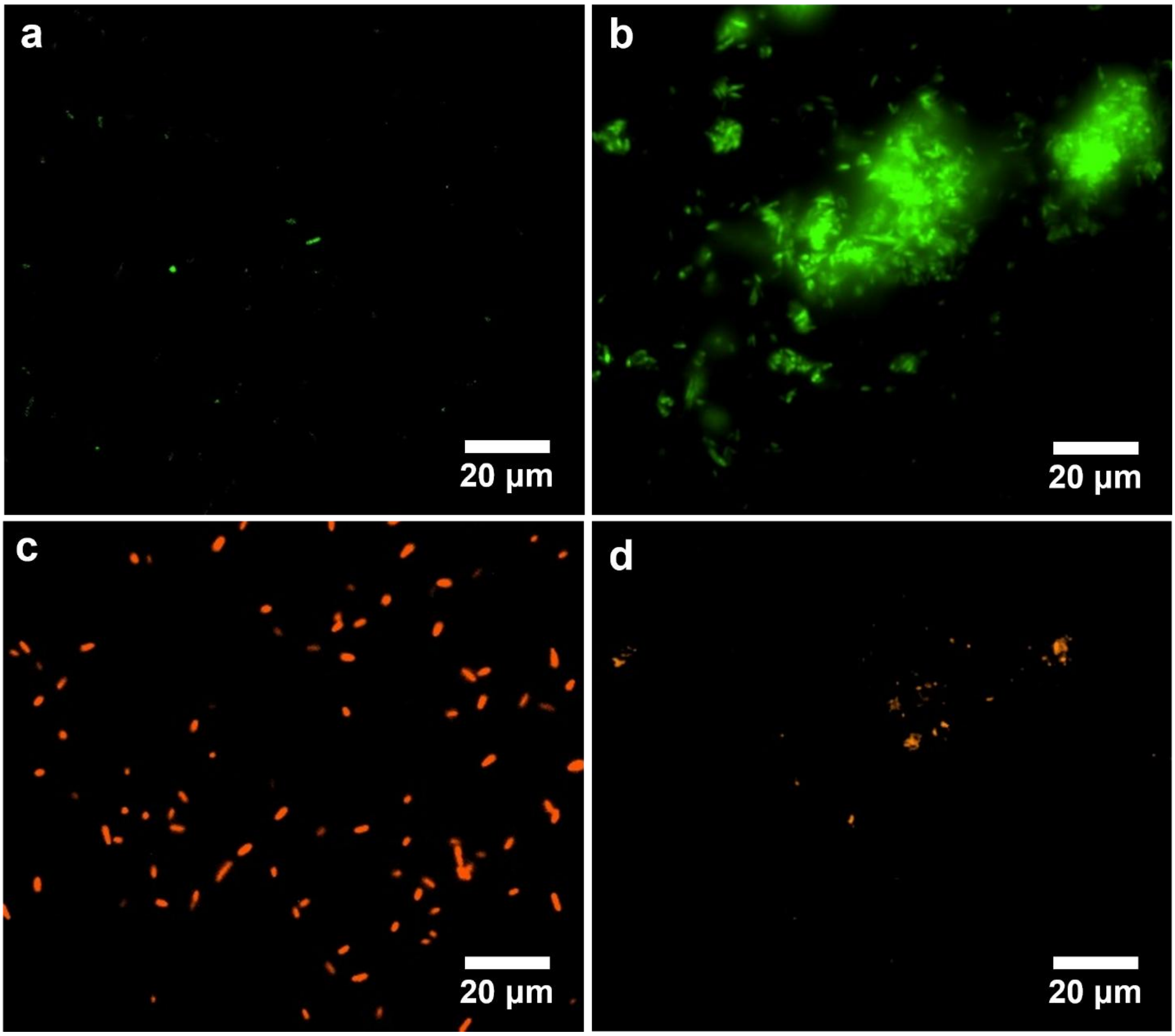

Figure S11. Live/Dead assay of native (a)(c) and PEI-CD (b)(d) cells after treatment with 5% acetonitrile.

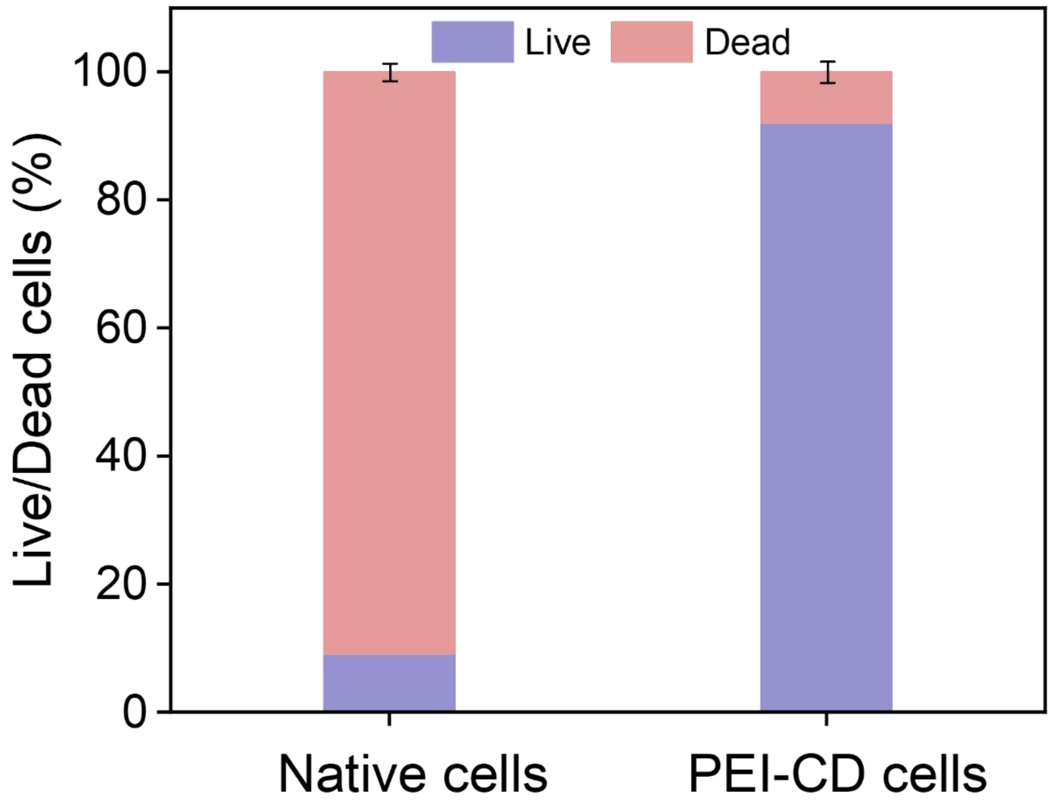

Figure S12. Quantification of live and dead cells from the microscopy assay in S9.

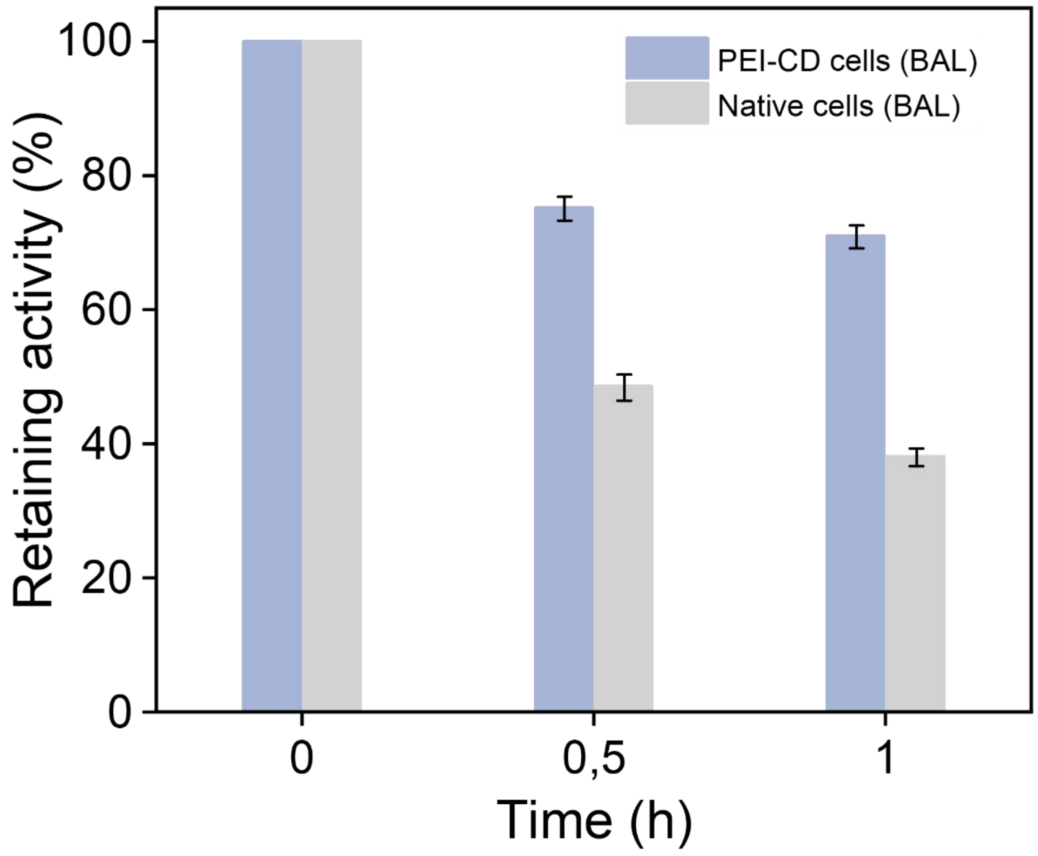

Figure S13. Enzymatic activity of BAL after interfacial stress.

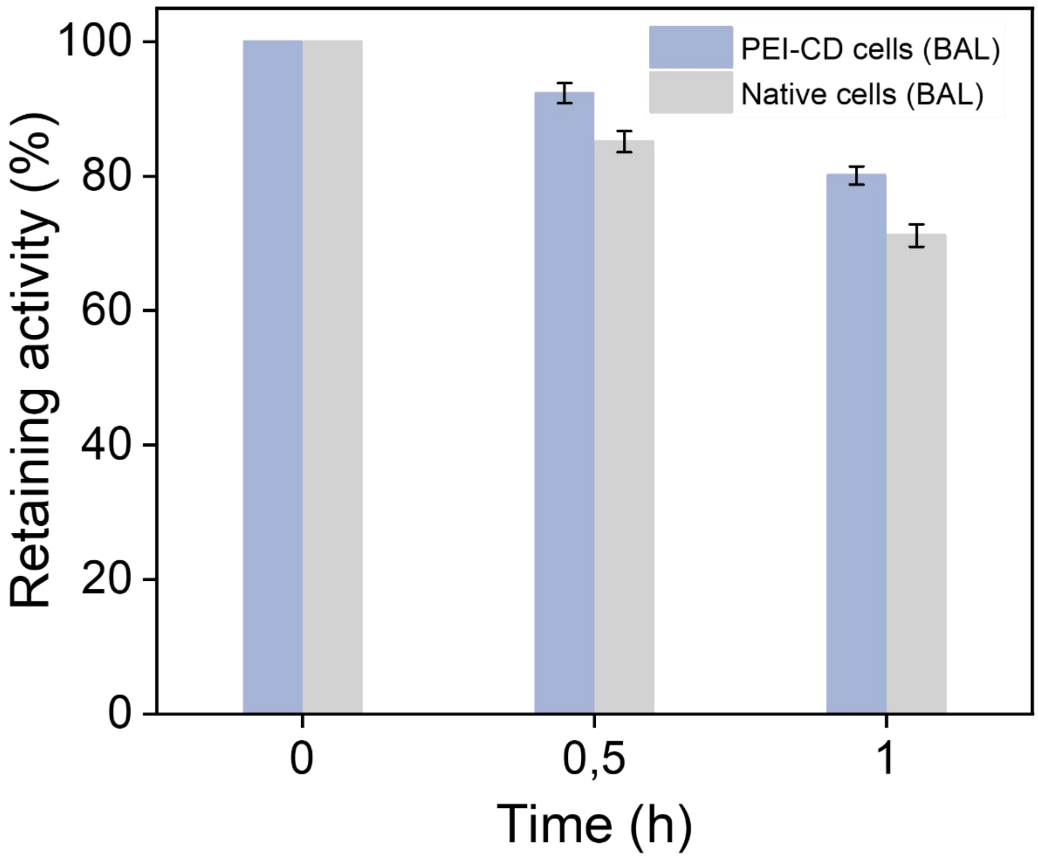

Figure S14. Enzymatic activity of BAL after treatment with 5% acetonitrile.

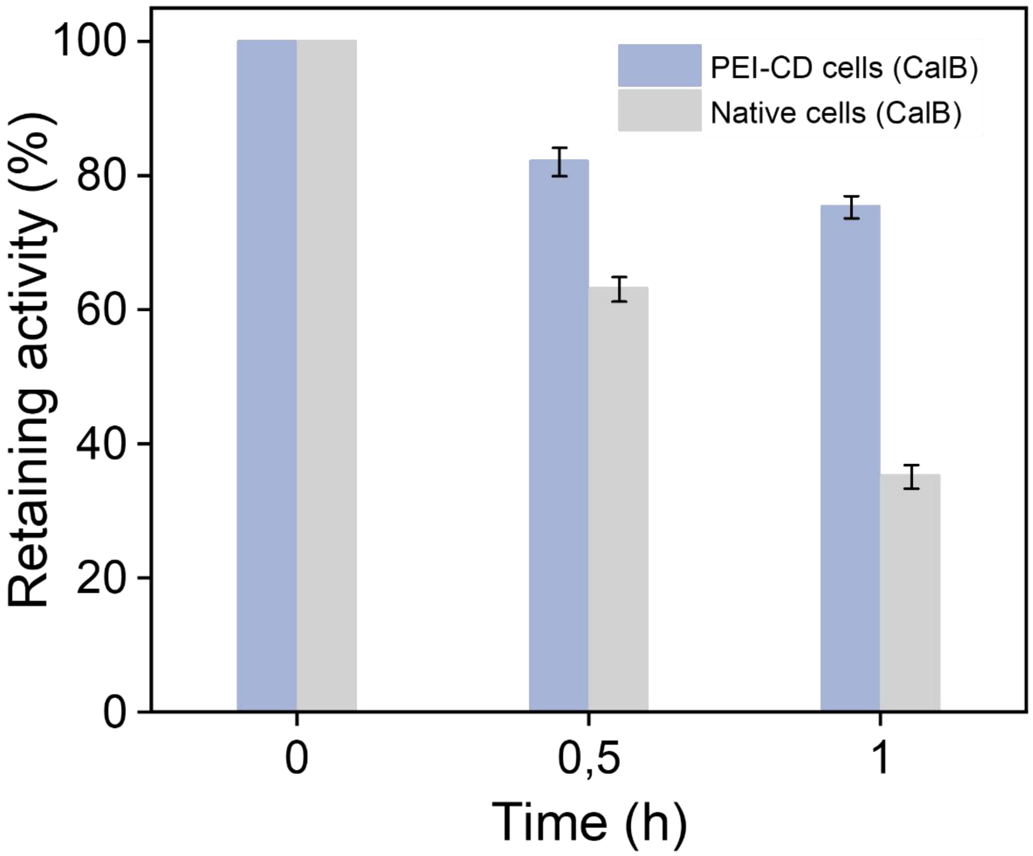

Figure S15. Enzymatic activity of CalB after interfacial stress.

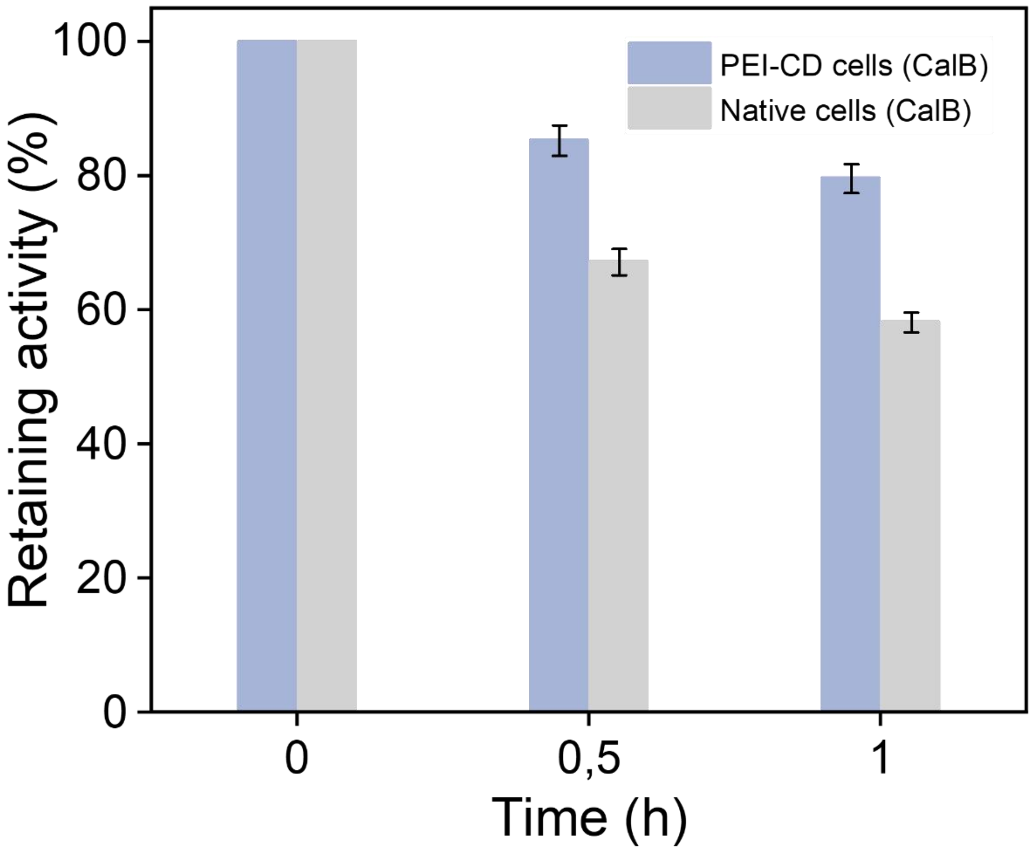

Figure S16. Enzymatic activity of CalB after treatment with 5% acetonitrile.

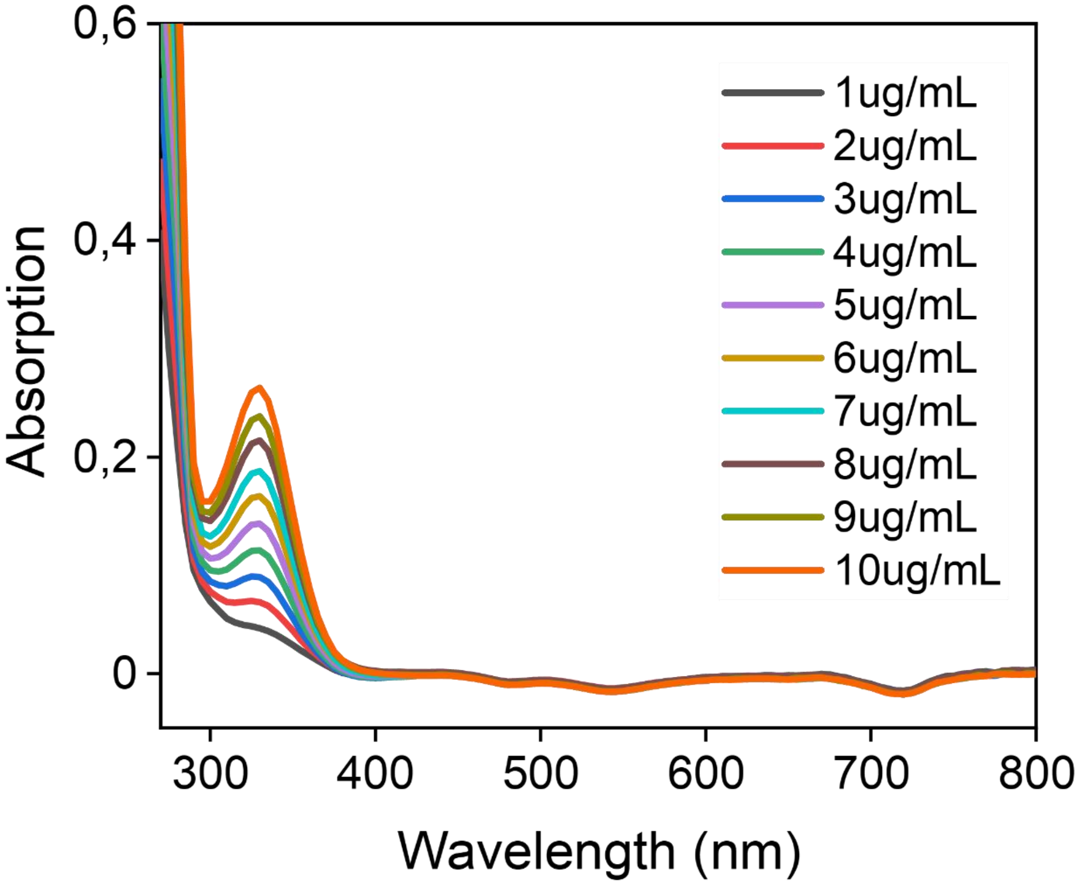

Figure S17. UV-Vis absorption spectra of AQS at different concentrations.

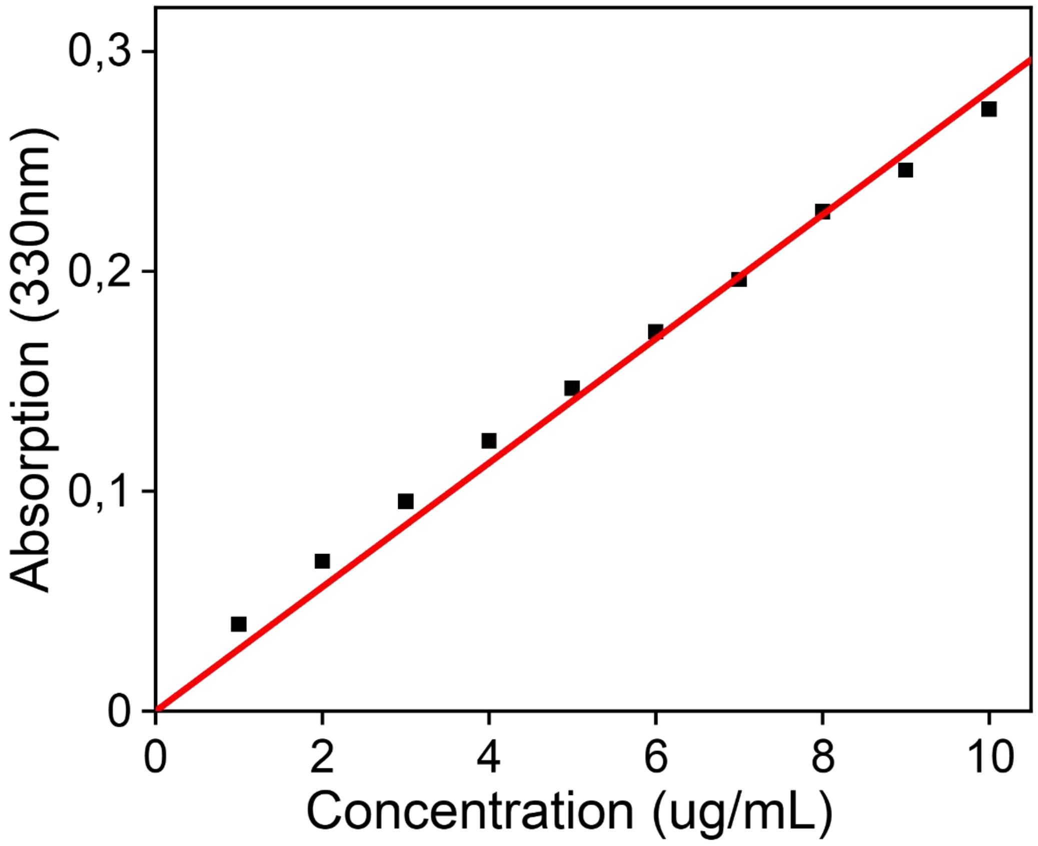

Figure S18. Standard calibration curve of AQS.

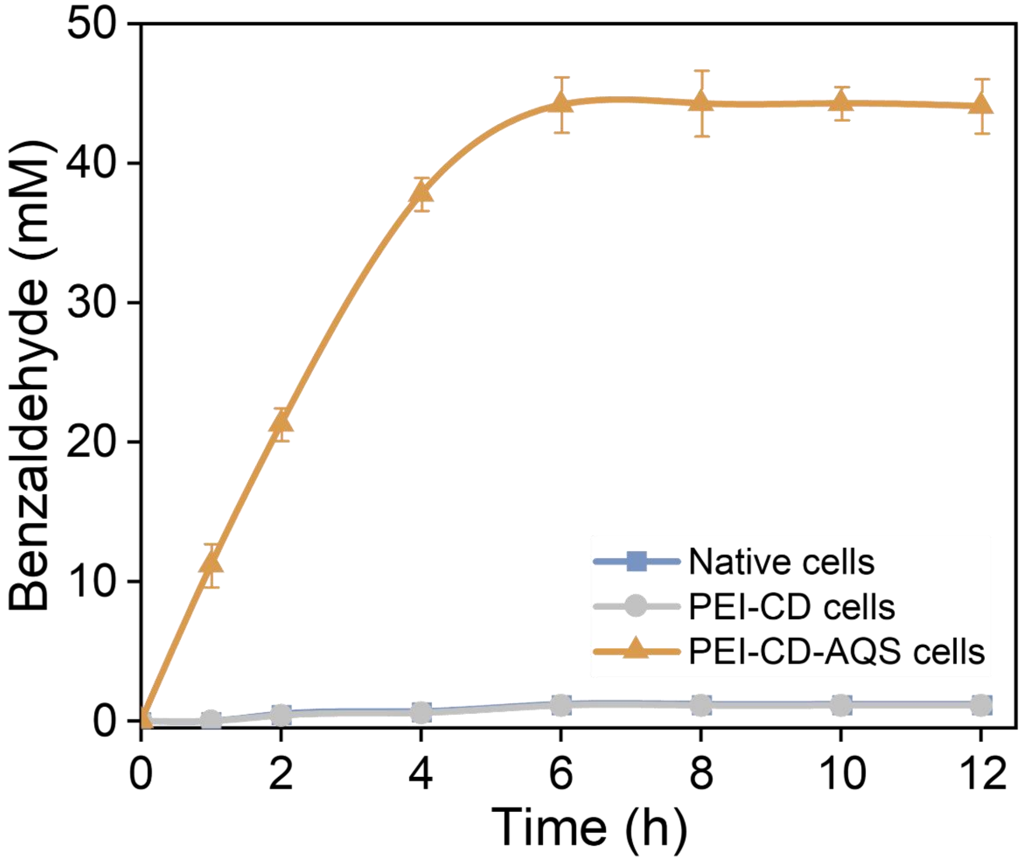

Figure S19. Evaluation of the catalytic activity of PEI-CD-AQS cells in the photooxidation of benzyl alcohol to benzaldehyde.

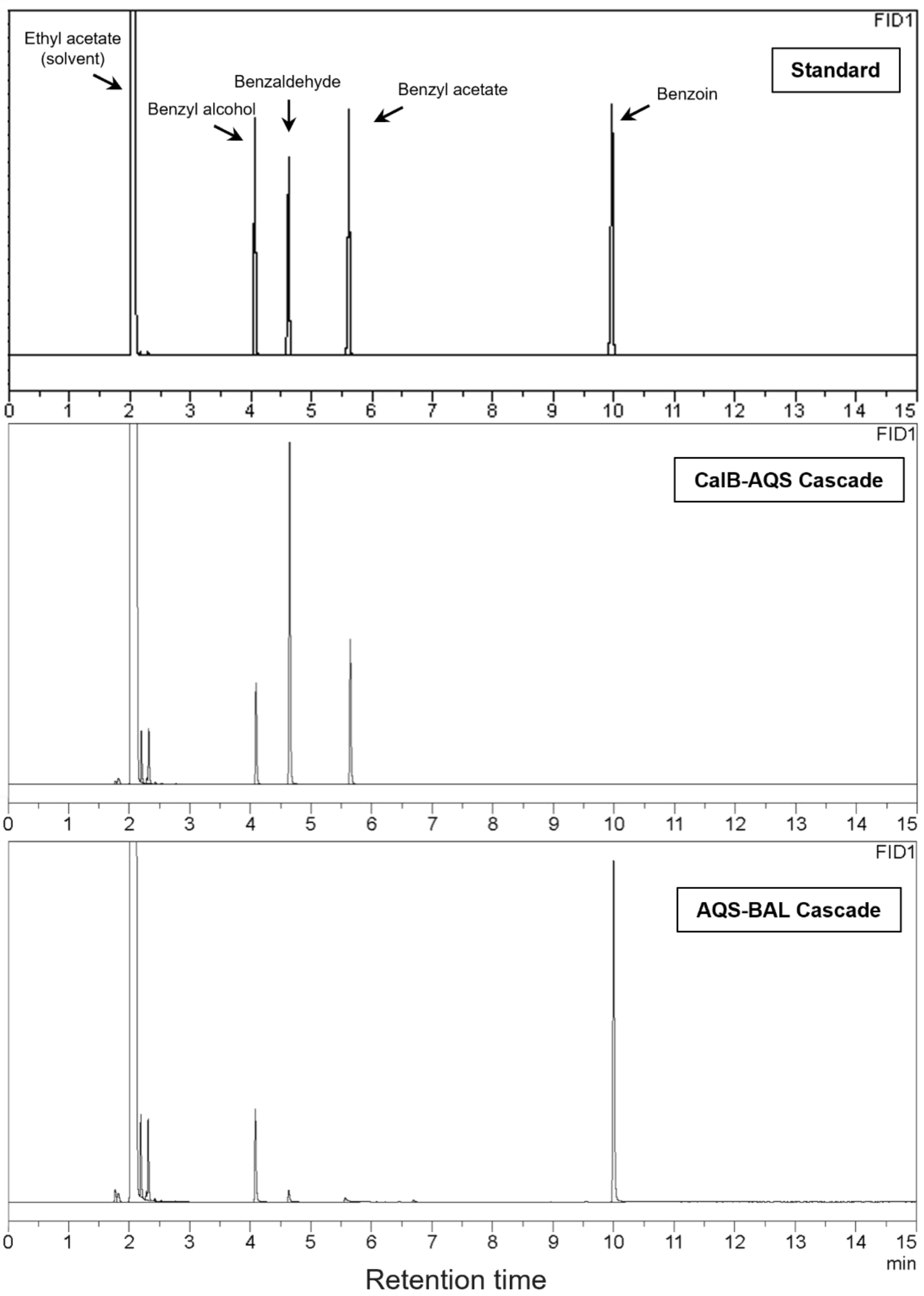

Figure S20. GC spectra of the standard chemicals and cascade reactions.

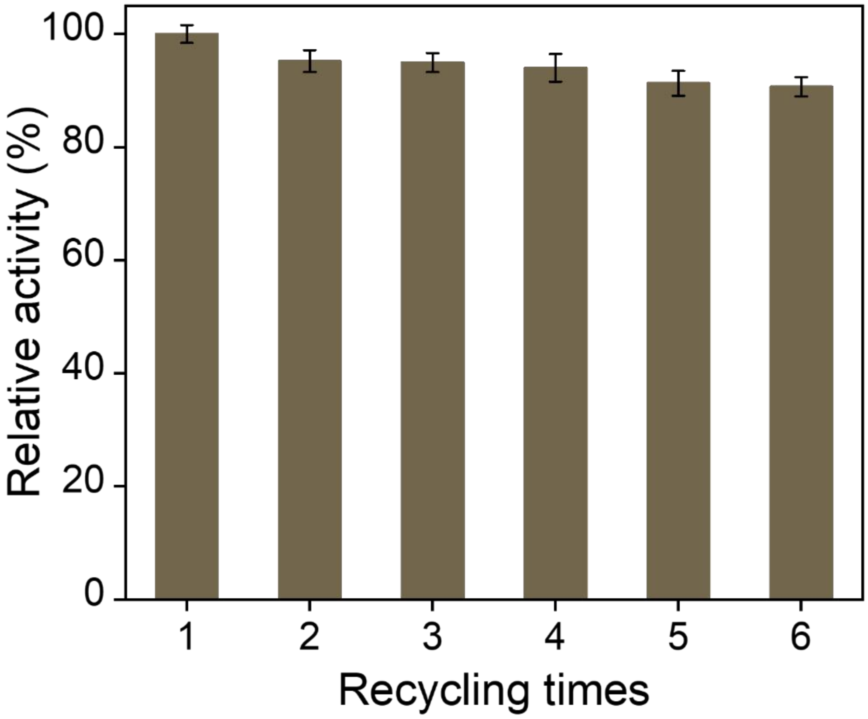

Figure S21. Recyclability of the PEI-CD-AQS cells (CalB) system.

Table S1. Calculated amount of AQS in PEI-CD cells at different concentrations of PEI-CD.

PEI-CD	30 mg	50 mg	80 mg	100 mg
AQS on PEI-CD cells	1.9 mg	3.2 mg	4 mg	4.2 mg

14. References

1 Y. Ping, Q. Hu, G. Tang and J. Li, *Biomaterials*, 2013, **34**, 6482.

2 Y. Xu, Y. Gao, L. Su, H. Wu, H. Tian, M. Zeng, C. Xu, X. Zhu and K. Liao, *Angew. Chem. Int. Ed.*, 2023, **62**, e202313638.

3 M. M. Kneen, I. D. Pogozheva, G. L. Kenyon and M. J. McLeish, *Biochim. Biophys. Acta. Proteins. Proteom.*, 2005, **1753**, 263.

4 F. W. Studier, *Protein. Expres. Purif.*, 2005, **41**, 207.
